# A Semi-Active Ankle Prosthesis With Automatic Transitions Between Passive and Active Modes

**DOI:** 10.1109/tmrb.2026.3713999

**Published:** 2026-07-16

**Authors:** Vishnu Aishwaryan Subra Mani, Connelly Ray Buchanan, Lukas Gabert, Tommaso Lenzi

**Affiliations:** Department of Mechanical Engineering, Utah Robotics Center, The University of Utah, Salt Lake City, UT 84112 USA; Department of Mechanical Engineering, Utah Robotics Center, The University of Utah, Salt Lake City, UT 84112 USA; Department of Mechanical Engineering, Utah Robotics Center, The University of Utah, Salt Lake City, UT 84112 USA; Department of Mechanical Engineering, Utah Robotics Center, and the Department of Biomedical Engineering, The University of Utah, Salt Lake City, UT 84112 USA, and also with the Rocky Mountain Center for Occupational and Environmental Health, Salt Lake City, UT 84111 USA.

**Keywords:** Prosthetics, series-elastic, actuators, actuator design

## Abstract

The most commonly prescribed ankle/foot prostheses are energy storage and return (ESR) devices, which have good performance in level ground walking, but have limited functionality in tasks requiring ankle mobility, such as stair ascent or descent. Powered prostheses aim to address this limitation by actively controlling the joint with actuators, but they tend to be heavier and taller and become unusable when the battery is depleted, hindering their clinical adoption. In this paper, we present a semi-active ankle prosthesis that integrates the energy storage and return characteristics of a passive prosthesis with the active joint articulation of a powered prosthesis. The proposed prosthesis combines a novel brake mechanism with a series elastic actuator to enable *Passive* and *Active* operation modes within a compact build height (141 mm) and lightweight (1.4 kg without batteries) form factor. Experimental validation with one lower-limb amputee demonstrates that the proposed prosthesis reproduces the gait dynamics of a passive prosthesis, independent of battery charge, in *Passive* mode, and matches the range of motion and actuation capabilities of a powered device during stair ascent and sit-to-stand transitions in *Active* mode. Automatic transitions between modes within a gait cycle allow for incline adaptation. Moreover, automatic transitions between gait cycles allow for seamless transitions between passive walking and active stair ambulation. This combination of functionalities has never been demonstrated in an ankle prosthesis and has the potential to improve mobility among the amputee population.

## I. Introduction

LOWER limb amputations pose significant challenges to mobility and quality of life due to disruptions in biomechanical function [1], [2]. The most common type of prosthesis for individuals with below-knee amputations is the energy storage and return (ESR) prosthesis [3], [4]. ESR prostheses use a composite structure without an actual ankle joint to store energy during rollover and release it during late stance, helping propel the user forward [5]. However, these lightweight devices are generally optimized for level-ground walking [6] and lack the capability to adapt to varying ground conditions, which requires a significant range of motion at the ankle [7]. As a result, ESR users often exhibit asymmetric gait patterns during stair ascent and descent [8], ramp ascent and descent [9], and sit-to-stand tasks [10].

To overcome these limitations, researchers have developed powered ankle prostheses that contain an actuator capable of actively controlling the position, impedance, or torque at the ankle joint [11], [12], [13], [14], [15]. Unlike passive devices, powered prostheses can contribute net positive energy, enabling active control of ankle joint position across variable terrain for toe clearance [16] and assisting users during stair ambulation by injecting or removing mechanical power during ascent and descent. However, including an actuator to perform all these actions tends to make powered prostheses heavier and taller than passive prostheses and requires substantial electrical energy to operate.

Powered ankle/foot prostheses commonly use advanced actuators like series-elastic actuators [14], [15], [16], [17] and polycentric actuators [18], [19]. These actuators achieve high torque and power and are highly backdrivable. However, they do not allow a user to ambulate when the battery is depleted. To address this problem, researchers have proposed parallel elastic actuators [20], [21], [22]. These actuators resist dorsiflexion via a unidirectional parallel spring, thereby allowing the prosthesis to support the user even when the battery is fully depleted. Parallel elastic actuators have enabled powered ankle/foot prostheses to reach the market [23]. However, this approach prevents the ankle joint from resting in a dorsiflexed position, as is common, for example, when climbing stairs or ramps or performing sit-to-stand transfers, because the motor must generate torque to counteract the spring in this configuration. Partly due to these limitations, powered ankle prostheses have not yet achieved widespread clinical success [24], [25].

To balance performance, build height, weight, and electrical energy consumption, researchers have explored semia-ctive ankle prostheses. These devices typically use a low-power actuator in combination with a spring or damper system [26], [27] to strategically deliver power during specific phases of the gait cycle. For example, swing-assist semi-active prostheses actively control the ankle joint position during swing, when the required ankle torque is low. In stance, they rely on their non-backdrivable transmission to support the user’s weight. As a result, users can walk even when the battery is fully depleted. Swing-assist prostheses have demonstrated improved toe clearance, comfort, and better adaptation to different terrain inclines [26], [28], [29]. However, they are unable to provide positive and negative power during stance as needed to adapt to different ambulation tasks, such as stair climbing and sit-to-stand transitions [30].

Variable-impedance semi-active prostheses use low-power electric actuators to modulate the ankle joint’s physical impedance (damping and stiffness) [31], [32]. Variable-damping prostheses are available on the market [33] and have shown improved foot adaptability and socket comfort, while providing adequate stability during quiet standing [34]. Variable-stiffness prostheses have not yet reached the market, but research studies have indicated the potential clinical benefits of varying joint stiffness for adaptation to slope and speed [27], [31]. Variable-impedance prostheses consume minimal electrical energy because the motor needs to perform only a small amount of work to change the joint’s mechanical impedance. Furthermore, these devices can continue to function reliably after battery depletion. However, they cannot provide net positive energy or actively control ankle joint position.

Underactuated mechanisms with mode switching have shown promising results in above-knee amputees performing varied ambulation activities [35], and below-knee devices could benefit from a similar implementation. For ankle prostheses, the ankle power requirements vary significantly between activities such as walking and ramp ambulation, compared to stair ambulation and sit-to-stand transitions. This paper introduces a semi-active ankle prosthesis that integrates an underactuated ankle-toe design using a series-elastic actuator and a novel brake mechanism. When the brake is disengaged, the device operates in *Active* mode and can deliver both positive and negative power as needed to support stair ambulation and sit-to-stand transitions. When the brake is engaged, the device operates in *Passive* mode, behaving like a passive ankle prosthesis and supporting the user’s weight even if the battery is depleted. The ankle can switch between Active and Passive modes during a gait cycle, allowing the user to navigate varying slopes more naturally. Additionally, switching modes between activities tends to maximize the device’s functional benefits during walking and stair ambulation. This paper presents the simulation, design, control, and benchtop testing of the semi-active ankle prosthesis. We also validate the proposed design and controllers with a single amputee participant ambulating on level ground, stairs, and ramps. By actively assisting stair climbing and sit-to-stand transitions while retaining the functionality of swing-assist devices, such as active swing repositioning and safe stance with depleted batteries, the proposed semi-active ankle/foot prosthesis could provide an alternative solution to improve mobility for individuals with lower-limb amputations.

## II. Design Objectives and Conceptual Design

### A. Design Objectives

The main goals of the proposed semi-active ankle prosthesis are to (1) function as a passive prosthesis during level-ground walking, (2) provide ankle articulation and power generation during stair ascent and sit-to-stand, and (3) actively adapt to ground incline in stance. To achieve passive walking, the prosthesis should be able to passively support an 80 kg individual during walking, which corresponds to a peak torque of 127 Nm at 10 deg dorsiflexion [36]. To support ambulation on stair ascent in active mode, the prosthesis should also provide a peak positive power of 1.75 W/kg [37], and an active range of motion of 11 degrees of dorsiflexion and 31 degrees of plantarflexion [38]. The brake should switch between locked and unlocked configurations in less than 300 ms, so that the prosthesis can transition between *Active* and *Passive* modes during swing when the user transitions from walking to stairs, or within the stance phase when the user walks on different inclines [39], [40]. Similar to market-available microprocessor-controlled ankles [33], [41], the proposed prosthesis should also have a maximum height of 142 mm and a weight of 1.5 kg (TABLE II). In addition, it should be compatible with standard prosthetic components, and all mechanical and electronic components should be enclosed, with no pinch points.

### B. Conceptual Design and Simulation

The proposed semi-active ankle prosthesis (Fig. 1) combines a novel disc brake with a series elastic actuator (SEA). In *Passive* mode, the brake is engaged, and the actuation mechanism functions as a non-linear passive spring. In *Active* mode, the brake is disengaged, and the actuation mechanism functions like a series elastic actuator. The proposed series-elastic actuator is implemented as a five-bar linkage (Fig. 2.D) with toe and ankle joints [15]. To design our semi-active ankle prosthesis, we used an iterative design approach based on a custom simulation framework in which we model and analyze three criteria: (i) the torque and speed capability of the series elastic actuator during ambulation in *Active* mode, (ii) the torque-angle curve of the ankle joint in *Passive* mode, and (iii) the holding torque and engaging time of the brake mechanism. This methodology builds on our prior work in actuator modeling and prosthesis design [15], [18].

#### 1) Active mode – Simulation:

In *Active* mode, the motor and transmission must generate the joint torque and power required for stair climbing (Fig. 2.A). To assess this capability, the simulation framework calculates the motor torque and power necessary to achieve physiological torque and angle profiles at the ankle joint [42], and check them against motor torque-speed limits based on the available supply voltage (Fig. 2.F). The required motor torque $\left(T_{motor}\right)$ is calculated using the dynamic equation shown in (1).

(1) $$T_{motor}=\frac{T_{a}}{TR\cdot \eta }+\left(I_{eq}\right)\cdot {\ddot{\theta }}_{motor}+\left(B_{eq}\right)\cdot {\dot{\theta }}_{motor}$$

where $T_{a}$ is the torque at the ankle, η is the eciency of the transmission, $I_{eq}$ is the inertia of the motor, $B_{eq}$ is the viscous damping at the motor, and ${\ddot{\theta }}_{motor}$ and ${\ddot{\theta }}_{motor}$ motor are the acceleration and velocity of the motor, respectively. The transmission ratio *T* *R* is calculated using the equation shown in (2).

(2) $$TR=N\cdot TR_{transmission}\cdot TR_{5bar}$$

where N is the reduction ratio of the gear, $TR_{transmission}$ is the reduction ratio of the transmission system, and $TR_{5bar}$ is the resultant transmission ratio from the kinematics. $TR_{5bar}$ is calculated by solving the kinematic chain R_1_R_2_P_1_R_3_R_4_ shown in Fig. 2.D [15].

(3) $$TR_{5bar}=\frac{T_{a}}{F}=\frac{\bar{OA}\cdot \bar{OC}\cdot \sin\left(\theta_{a}\right)}{\bar{AC}}$$

where F is the spring force, $\theta_{a}$ is the ankle angle Fig. 2.C, and $\bar{OA}$, $\bar{OC}$, and $\bar{AC}$ are defined as shown in Fig. 2.D.

The motor velocity is defined using equation (4), which is calculated using the ankle velocity and transforming the linear rate of spring deflection into rotational velocity using $TR_{5bar}$.

(4) $${\dot{\theta }}_{motor}=TR\cdot {\dot{\theta }}_{a}-N\cdot TR_{transmission}\cdot \frac{\dot{F}}{K}$$

where ${\dot{\theta }}_{a}$ is the velocity of ankle, $\dot{F}$ is the spring force rate and K is the spring stiffness. Fig. 2.F shows the output of the simulation during a stair ascent stride.

#### 2) Passive mode – Simulation:

In *Passive* mode, we assessed the ankle torque-angle curve during ambulation [43], [44], as a function of the five-bar linkage dimensions di> (R_1_R_2_R_3_P_1_R_4_) in an underactuated configuration. Since the actuation mechanism functions like a series spring, the mathematical definition of the torque-angle relationship at the ankle can be simplified to (5).

(5) $$\tau_{\text{a}}=k_{s,torsion}\cdot \theta_{\text{a}}$$

where $\tau_{\text{a}}$ is the ankle joint torque, $k_{s,torsion}$ is the equivalent torsional stiffness at the ankle joint, and $\theta_{\text{a}}$ is the ankle angle. We can define $k_{s,torsion}\cdot \theta_{\text{a}}$ as a function of the stiffness of the linear spring $k_{s}$, the distance between points $\bar{OA}$, the deflection of linear spring $\text{Δ}\bar{AC}$, and the angle of the segment $\bar{OA}$ with respect to the ankle joint $\left(\psi_{a}\right)$ as shown in (6).

(6) $$k_{s,torsion}\cdot \theta_{\text{a}}=k_{s}\cdot \text{Δ}\bar{AC}\cdot \bar{OA}\cdot \sin\left(\psi_{a}\right)$$

As simulation inputs, we used the ankle joint positions from a biomechanical dataset of healthy participants [36] and different spring stiffnesses, assuming a set equilibrium ankle angle of 0 degrees. We then selected the spring stiffness that matched the non-disabled torque-angle curves to achieve the same peak torque, as shown in Fig. 2.G.

#### 3) Brake – Simulation:

The brake mechanism acts directly on the motor shaft and consists of a brake disk actuated by a cam-and-piston mechanism, driven by a non-backdrivable worm drive powered by a miniaturized electric motor (Fig. 3). We developed a model of the brake mechanism to dimension its components and ensure they provide the required holding torque at the motor shaft (Fig. 3.A). First, we model the axial force on the brake pistons $\text{(}F_{a}\text{)}$, restoring spring force $\text{(}F_{r}\text{)}$ as a function of holding torque $\tau_{h}$, friction coefficient $\mu_{\text{f}}$, and the mean radius of the pad ${\text{r}}_{p}$ as shown in (7) and (Fig. 3.B).

(7) $$F_{a}-F_{r}=\frac{\tau_{h}}{2\cdot \mu_{f}\cdot r_{p}}$$

Then, we model the rotation of the cam $\left(\theta_{c}\right)$ to the linear displacement $\left(\delta_{y}\right)$ of the piston acting on the brake disk to generate the braking force $F_{a}$. As shown in (8), the displacement of the piston $\delta_{y}$ is a function of the radius of the cam $\left(r_{c}\right)$ and the offset angle (β), which is the angle at which the cam is in contact with the piston, as shown below.

(8) $$\theta_{c}=\sin^{-1}\left(\frac{\delta_{y}}{r_{c}}\right)-\beta$$

Finally, we can relate the cam torque $\left(\tau_{c}\right)$ to the piston force $\left(F_{a}\right)$:

(9) $$\tau_{c}=F_{a}\cdot r_{c}\cdot \cos\left(\beta +\theta_{c}\right)$$

The brake motor must deliver a torque equal to or greater than $\tau_{\text{c}}$ to generate the required axial force $F_{a}$ at the piston. Thus, the torque at the brake motor $\tau_{b}$ must be

(10) $$\tau_{b}=\frac{\tau_{c}}{n_{b}\cdot n_{w}}$$

where $n_{b}$ is the primary reduction ratio after the motor and the $n_{w}$ is the reduction ratio of the worm drive. In addition, we model the brake-engaging time $\left(t_{b}\right)$ as a function of the brake motor speed $\left(\omega_{b}\right)$, as stated in (11).

(11) $$\omega_{b}=n_{b}\cdot n_{w}\cdot \frac{\theta_{c}}{t_{b}}$$

Using this model, we developed a brake motor mechanism that fits within the desired size profile of the proposed ankle prosthesis and has a sufficiently short engagement time.

## III. Actuation and Mechanical Structure

The semi-active ankle comprises three main structural components (Fig. 4.A): a shank frame, a foot frame, and a toe frame. The shank frame is machined out of 7075 aluminum, and it integrates a custom instrumented pyramid adapter for ground reaction force sensing [45]. It connects to the foot frame through the main ankle joint shaft. The foot frame is made of carbon fiber and is sourced from a commercially available variable-impedance prosthesis (Meridium, Ottobock) [33], [41]. The toe frame is custom-machined from 7075 aluminum and connects to the foot frame via the toe joint shaft. The structural components enclose the series-elastic actuator, the brake mechanism, and the electronic system.

The series-elastic actuator comprises a brushless motor (Maxon EC22, 4-pole, 24 V, 90 W) coupled to a 2.52:1 helical gear reducer (Boston Gear). The helical gear reducer drives a ball screw (Ewellix 12×2R SD), which is held by an angular contact ball bearing (ZKLN0619). The ball nut threads into a custom-machined spring-capture mechanism that holds a steel coil spring (McMaster 1804N218) [46]. The opposing side of the spring capture mechanism attaches to the toe frame through a shaft and includes passive degrees of freedom that minimize mechanical constraints on the actuation.

The brake mechanism is connected to the motor powering the series-elastic actuator via a custom mounting plate. It comprises a brushless motor (Maxon EC10, 18 V, 8 W) coupled to a 16:1 planetary gearbox (Maxon GP10 16:1). This motor/reducer drives a 0.4-module worm drive. The worm gear is press-fit to a camshaft, upon which rests the brake piston. The disc brake comprises a fixed brake pad attached to the main motor frame, a brake disc mated to the main motor shaft via a hexagonal pattern to allow axial movement, and a brake pad actuated by the piston. Springs are positioned between the fixed brake pad and the actuated brake pad to ensure that the brake disengages when the force from the brake piston is removed. At the disengaged position, the gap between the brake disc and brake pads allows the brushless motor (Maxon EC22, 4-pole, 24 V, 90 W) to rotate freely. As the brake engages, the linear displacement of the piston axially moves the actuated brake pad into the floating brake disc, which in turn moves against the fixed brake pad, arresting the brushless motor rotation. The brake mechanism is shown in detail in Supplementary Fig.S1.

The proposed semi-active ankle prosthesis weighs 1.4 kg without batteries and is primarily intended for use with an active knee prosthesis. The final assembly achieves a total ankle range of motion of 49.5° (23.7° of plantarflexion and 25.8° of dorsiflexion) and a toe flexion of 45°. All mechanical and electrical systems, except for the batteries, are enclosed in the device, which is 261 mm long, 70 mm wide, and 141 mm tall.

## IV. Embedded Electronics and Sensing

The series-elastic actuator motor is powered by a high-power current driver (G-TWIR 80/80SES, Elmo) that implements field-oriented control based on a 12-bit absolute encoder (RLS RM08) mounted on the motor shaft. A smaller current driver (Maxon Escon Module 24/2) drives the brake motor using sinusoidal commutation via hall sensors integrated in the brake motor.

The ankle and toe joints are equipped with absolute magnetic encoders (AMS AS5047U). The absolute position of the camshaft in the brake mechanism is measured using a rotary encoder with linear voltage output (RLS RM08VC) mounted at the end of the camshaft. A microcontroller (PIC32MK0512MCF100) reads data from all encoders at 1 kHz and applies a 15 Hz first-order Butterworth lowpass filter. A second microcontroller (PIC32MK0512MCF100) receives the filtered data and runs the mid-level control algorithms at 715 Hz. The second microcontroller also communicates with current drivers for both the joint and brake motors, using differential PWM signals.

The axial ground reaction force and sagittal torque are measured by a custom ground reaction force sensor integrated in the male pyramid adapter [45]. The spring deflection is determined by combining the joint angles measured from the absolute joint encoders and the ballscrew nut position estimated by the series-elastic motor encoder. The ankle torque is determined by multiplying the spring stiffness by the spring deflection to obtain the spring force, and then multiplying that force by its moment arm with respect to the ankle joint (6).

For this study, the semi-active ankle was used in conjunction with a powered knee prosthesis [47], which housed an 8-cell 1200 mAh LiPo battery (33.6 V), to power both the ankle and knee actuators, and a Raspberry Pi to run high-level control routines and record data at 500 Hz. The Raspberry Pi also communicated with an external laptop via Wi-Fi to enable data visualization and tuning of control parameters. A microcontroller in the knee relays high-level control commands from the Raspberry Pi to the semi-active ankle microcontrollers, running at 715 Hz. All sensor processing and mid-level control commands are executed within the ankle microcontrollers. The electronics architecture is shown in Supplementary Fig.S2.

## V. Controller

The semi-active ankle prosthesis has two modes of operation: Active Mode and Passive Mode. In *Passive* mode, the semi-active ankle functions as a passive energy-storage-and-return prosthesis. In this mode, the brake is engaged, effectively locking the ballscrew, so that moving the ankle from its resting position requires compressing or extending the SEA spring. Because the brake mechanism is non-backdrivable and the SEA motor is disabled, no electrical energy is needed in this mode. In *Active* mode, the semi-active ankle functions as a powered ankle prosthesis. The brake is disengaged, allowing the ballscrew to move, and the SEA motor is enabled to provide the desired torque at the ankle joint. In this mode, the ankle joint is controlled in impedance during stance and in position during swing, as determined by a finite-state machine based on the GRF sensor.

When the *terrain-adaptation controller* is active, the brake automatically engages and disengages within each step, enabling the prosthesis to actively conform to the ground slope in early stance while passively rolling and pushing off in mid and late stance. This transition follows a finite-state machine (Fig. 5.B). At heel strike, the brake is disengaged, and the ankle is in impedance control, allowing the foot to conform to the ground, similar to an active ankle/foot prosthesis [48]. When the shank angle becomes greater than a set threshold $\left(\theta_{Shank}^{thresh}\right)$, the brake engages, essentially setting the equilibrium angle of the ankle for rollover and pushoff. At toe-off, the brake disengages, and the ankle returns to a neutral position through closed-loop PID position control. This controller enables the semi-active ankle to actively conform to the ground, although it does not provide active pushoff power.

When the *walk-stair transition controller* is active, the device automatically switches between Active and Passive modes as the user transitions between stairs and level-ground walking. The transition between active and passive modes occurs only in swing and depends on the knee and thigh angles, which define the $K^{Syn}$ control parameter, as detailed in [48]. As shown in Fig. 5.A, if $K^{Syn}$ is 0, the brake engages, and the device enters *Passive* mode (walking). If $K^{Syn}$ is 1, the brake disengages, and the device enters *Active* mode (stairs). In active mode, the virtual ankle equilibrium angle changes from an initially dorsiflexed to a neutral position as the knee joint extends during stair ascent. In swing, the ankle initially plantarflexes to clear the step and then dorsiflexes to allow proper placement of the foot on the next stair. A detailed implementation of the controller is described in [48] and summarized in Supplementary Fig.S4 and Fig.S5. The brake position control uses a proportional-derivative (PD) regulator to control the camshaft angle. The brake is engaged when the camshaft position is 58° and disengaged when the camshaft position is 0°. After 400 ms from the engagement or disengagement command, the brake motor driver is disabled to conserve power. The mid-level controller disables the SEA motor when the brake is engaged and enables it when the brake is disengaged, thereby switching between Active and Passive Modes.

When the sit-to-stand controller is active, the device defaults to Active mode operation. The commanded torque at the ankle is the sum of virtual stiffness $T_{Ankle}^{Stiffness}$ and virtual damping torques $T_{Ankle}^{Damping}$ where the former term is calculated by the difference between current ankle angle $\theta_{Ankle}^{Meas}$ and desired equilibrium angle $\theta^{eq}$ and the latter term is a dynamic function of hip velocity $v_{Hip}^{Meas}$ allowing for biomimetic energy injection as stated in [48].

## VI. Experimental Validation

### A. Benchtop Testing

To characterize the actuator’s output impedance, we connected the shank frame to a bench through a 6-axis force-torque sensor (Sunrise Instruments, M3713D). Then, we manually moved the foot segment following a sinusoidal pattern at frequencies from 0.25 Hz to 1.75 Hz over 115 seconds, while we recorded the ankle velocity and the ankle torque estimated by the external sensor (Fig. 6.A). Through a frequency transformation of the data, we calculate the actuator’s mechanical impedance to vary between 2.9 Nms/rad and 5.9 Nms/rad for the tested frequencies (Fig. 6.B).

To assess the PID controller’s ability to track a desired position, we performed a step-response test, commanding three position steps (5°, 10°, 15°) from an ankle position of 0°, each repeated 5 times. The rise time across the three conditions ranged from 76 ms to 80 ms (Fig. 6.C), yielding a −3-dB bandwidth of 4–5 Hz, which is within the bandwidth required for ambulation [49].

To characterize the open-loop torque controller, we mounted the semi-active ankle between a 6-axis force-torque sensor (Sunrise Instruments, M3713D) and an aluminum frame. Then, we commanded torque steps of 20 Nm and 40 Nm in both plantarflexion and dorsiflexion, repeating each condition 5 times. In plantarflexion, the rise time was 28 ms and 38 ms, and the overshoot was 64% and 79%, for the 20-Nm and the 40-Nm step, respectively (Fig. 6.D). In dorsiflexion, the rise time was 26 ms and 32.5 ms, and the overshoot was 50% and 57% for the 20-Nm and 40-Nm steps, respectively. Across conditions, the steady state error was less than 10% of the commanded value. Based on the measured rise times, the −3-dB bandwidth ranged between 9.2 Hz and 13.5 Hz.

To verify the stiffness of the series elastic spring, we mounted the linear spring and capturing mechanisms to a linear testing machine equipped with a 50 kN load cell (Instron 68TM-50, 2850–50KN). The testing machine applied a linear ramp of force from 0 kN to 2 kN at a rate of 4 kN/min for 5 cycles in tension and compression, while we measured the spring’s deflection (Fig. 6.E). Using linear regression, we estimated the tension and compression stiffnesses to be 633 N/mm and 961 N/mm, respectively.

We assessed the closed-loop position control performance of the brake mechanism using a step-response test. Specifically, we commanded a position step from 0° to 58°, representing the fully disengaged-to-fully-engaged brake configuration. We repeated the test 5 times to estimate the controller’s rise time and −3-dB bandwidth, which were 214 ms (Fig. 6.F) and 1.6 Hz, respectively. Notably, the rise time corresponds to the brake engagement time.

To validate the maximum holding torque of the brake mechanism, we applied an external torque with the brake engaged and the ankle angle at 0°. To accurately measure external torque, we mounted a 6-axis force-torque sensor (Sunrise Instruments, M3713D) to the shank frame of the semi-active ankle. We manually applied up to 200 Nm of torque while rigidly constraining the foot to the ground twice. Results of this test show that the brake consistently holds more than 150 Nm (Fig. 6.H), which is more than the target holding torque (TABLE I).

### B. Amputee Testing

We recruited one individual with above-knee amputation (male, 30 years, 72.5 Kg) to test the proposed semi-active ankle and related control modes (Fig. 7) built on our previously validated high-level controllers used with a powered knee prosthesis [15], [18]. This experiment was approved by the University of Utah Institutional Review Board (protocol 00103197). Before participation, the user provided written informed consent, including consent to the publication of photographs and videos of the experiments. We conducted the validation tests in two sessions. During the first session, the user spent two hours familiarizing themselves with the semi-active ankle and its related control modes. During the second session, we collected data for all control modes.

To test *Passive* mode, the participant walked on a 15-m level-ground pathway five times at a self-selected speed. Before starting the test, the experimenter switched the high-level controller into *Passive* mode while the user was standing, engaging the brake at a neutral standing angle. We recorded ankle joint angle and torque using onboard sensors in the semi-active prosthesis (Fig. 8.A), and compared them to non-amputee data from an available dataset [36], and to data obtained from the same participant using their prescribed passive ESR foot [4], which was recorded in a previous session using the marker set for full motion capture as described in [50]. With the semi-active ankle, the user achieved a peak dorsiflexion angle of 5.2° and a peak plantarflexion torque of 128 Nm. With the prescribed prosthesis, the user achieved a peak dorsiflexion angle of 12.8° and a peak plantarflexion torque of 99 Nm. Comparison with non-amputee data shows that the two devices achieve similar normative ankle angles (9.8 degrees of dorsiflexion) and plantarflexion torque (117 Nm).

To test the *Active* mode, the experimenter switched the high-level controller into *Active* mode while the user was standing. The participant ascended and descended a seven-step staircase five times with the semi-active ankle and with a powered ankle [15], [48]. In addition, the participant performed 5 sit-to-stand transitions. We recorded ankle joint angle and torque using onboard sensors in both the semi-active and powered ankle prostheses and compared them to data from an available non-amputee dataset [37] for stair ascent and descent and for sit-to-stand transition [51] (Fig. 8.B). In stair ascent, the peak dorsiflexion angle was 19.2° for the semi-active ankle and −22.8° for the fully powered ankle. In stair ascent, the peak torque in stance was 29.0 Nm and 32.4 Nm for the semi-active and fully powered ankles, respectively. In stair descent, the peak swing phase plantarflexion angles were 19.2° for the semi-active ankle and 17.8° for the fully powered ankle. The peak dorsiflexion angles during stance were 21° and 22° for the semi-active and fully powered ankles, respectively. In stair descent, the peak torque was 42.4 Nm and 55 Nm for the semi-active and fully powered ankles, respectively. In the sit-to-stand transitions, the peak dorsiflexion angle was 17°, and the peak torque was 24 Nm for the semi-active ankle. The non-disabled dataset reached 26.2° of dorsiflexion and 27 Nm of peak torque.

To test automatic transition during *walk-stair*, the participant walked 2 m to the staircase, ascended it, and then walked another 2 m. During walking, the ankle was in *Passive* mode, the brake was engaged, and the spring provided support for ambulation (Fig. 8.C), similar to the level-ground walking test (Fig. 8.A). As the participant transitioned from walking to ascending stairs, the synergy gain increased from zero to one (Fig. 8.B), and the controller disengaged the brake mechanism, changing the brake angle from 58° to 0° (Fig. 8.B). After the brake mechanism disengaged, the SEA motor was enabled, and the semi-active ankle behaved similarly to the Stair Ascent test (Fig. 8.C). As the participant transitioned from ascending stairs to walking, the synergy gain decreased from 1 to 0 (Fig. 8.B), the brake mechanism engaged, changing the brake angle from 0° to 58°, the SEA was disabled, and the user walked in *Passive* mode.

To test automatic transition during terrain adaptation, the participant ambulated in three scenarios: ramp ascent (11°), level-ground walking, and ramp descent (11°). The length of the ramp and level ground was 6 m. Before data collection, we tuned the threshold parameters for the minimum shank angle and time after contact to identify a suitable set of values. The *minimum shank angle threshold* was set to −15°, and the *time after contact threshold* was set to 0.1 s. Fig. 8.D shows the brake angle, ankle angle, torque, and shank angle averaged over 8 strides for ramp ascent, level ground walking, and rampdescent. The motor torque in early stance peaked at −13.7 Nm for ramp ascent, −18.2 Nm for walking, and −16.6 Nm for ramp descent. The brake engaged at 46% of stride during ramp ascent, 27% during walking, and 30% during ramp descent. The brake engaged at an ankle angle of −13.3° in ramp ascent: 2.88° in walking, and 0.44° in ramp descent. The maximum torque in late stance was 104 Nm in ramp ascent, 123.5 Nm in walking, and 101.5 Nm in ramp descent. At toe-off, the device returned to *Active* mode, and the SEA motor returned the ankle to an equilibrium of 0°.

## VII. Comparative Analysis

We compare the proposed semi-active prosthesis with other powered, semi-active, and passive ankle devices (TABLE II). Compared to most powered devices, the semi-active ankle is lightweight (1.4 kg without batteries). With an additional weight of 216 g for batteries (an 8-cell 1200 mAh lithium polymer battery) and covers, the proposed ankle would weigh 1.6 kg, which is similar to market-available variable-impedance devices such as the Ottobock Meridium (1.5 Kg) and semi-active ankle like the Ossur Proprio (1.5 Kg). The proposed brake mechanism weighs less than 100 g, indicating that clutching action under high loads can be achieved with only a slight (5–7 %) increase in the system’s overall weight. Furthermore, the brake is designed to prevent the motor from moving under load rather than to dissipate mechanical energy. By locking the motor, the brake allows the series spring to store and release energy. Thus, in passive mode, the proposed semi-active ankle behaves more like a passive ESR prosthesis than a controllable damper ankle, which primarily dissipates energy. Moreover, in active mode, the motor can act as a damper, partially regenerating mechanical energy during negative-power operations.

The active range of motion of the semi-active ankle (−26°/ + 24°)is slightly larger than that of the Utah Bionic Leg Ankle (−20°/ + 20), and a much larger than that of the Ottobock Empower (0/ + 22°).The semi-active ankle has a high dorsiflexion angle (26°) compared to most semia-ctive devices, such as Bartlett (12°), the Non-backdrivable RAP (20°) and variable-impedance devices such as Ottobock Meridium (14.5°).

It is worth noting that the active motor power of the proposed semi-active ankle (115 W) is substantially higher than that of most semi-active ankle prostheses, which generally have motors rated at less than 20 W. The only other semi-active ankle with the same level of active motor power is the AMP Foot 4 + (200 W). However, the AMP Foot 4+ is designed to provide full power for all activities and thus weighs 2.3 Kg without the batteries. The proposed semi-active ankle uses a smaller motor than those used in other powered ankle/foot prostheses, such as the Utah Bionic Leg Ankle and Utah Polycentric Ankle (TABLE II). Thus, due to thermal limitations, it cannot sustain the same power injection level in active mode.

The proposed semi-active ankle has a height of 141 mm, which is less than most powered, semi-active, and passive prostheses (TABLE II). The only three devices with a smaller build height are the Utah polycentric ankle [18], the VSPA [27] and the VSF [31]. The Utah polycentric ankle employs a seven-bar mechanism with a moving instantaneous center of rotation, resulting in a low-profile system measuring 120 mm in height. However, the Utah polycentric ankle, like most other powered devices (TABLE II), cannot support the user’s weight when the battery is depleted. While not currently implemented, the semi-active ankle can monitor the battery voltage, return the ankle to a neutral position, and engage the brake before the battery is fully depleted, thereby allowing safe passive operation across different ambulation activities (See Supplementary Video). The VSPA uses a cam at the joint, enabling a small build height of 118 mm and supporting the user’s weight when the battery is depleted. However, the VSPA lacks the capability to actively change the ankle position. The VSF uses a sliding mechanism along the footplate to adjust forefoot stiffness, allowing a build height of 87 mm. However, the VSF cannot actively inject or remove power during gait.

## VIII. Discussion

Semi-active prostheses aim to balance function with weight/build height, offering an alternative to fully powered and passive options. Most semi-active ankle prostheses focus on adjusting physical impedance (e.g., damping, stiffness) or controlling ankle joint position during the swing phase. In contrast, our semi-active design provides moderate power injection and absorption during both stance and swing phases, enabling a more natural gait during stair ascent/descent (*Active Mode*) and on inclined terrain (*Terrain-Adaptation controller*). Additionally, by locking the ankle joint with the proposed brake system (*Passive Mode*), our semi-active ankle can function as a passive prosthesis, storing and returning energy during gait without using electrical power. The proposed semi-active ankle prototype weighs 1.4 kg and is 141 mm tall, comparable to other semi-active ankles with variable impedance or swing-only repositioning capabilities. This paper demonstrates, for the first time, that combining swing and stance adaptability through a series-elastic actuator and a brake mechanism creates a viable semi-active ankle prosthesis with unique, innovative features compared to earlier devices.

The proposed semi-active ankle prosthesis can partly function as a powered prosthesis during stair ascent, descent, and sit-to-stand transitions [52], [53]. During stair ascent, the semi-active ankle actively dorsiflexes in swing, enabling the user to position their center of mass over the foot at the beginning of stance [42], [54]. In stance, the semi-active ankle actively plantarflexes, helping the user to extend the knee. In contrast, during stair descent, the semi-active ankle plantarflexes in swing to point the toe down. In stance, it actively generates plantarflexion torque to slow the descent. In sit-to-stand transitions, the ankle is dorsiflexed at the beginning, allowing the user to position their center of mass over the foot. However, unlike many fully powered prostheses [26], the semi-active ankle cannot provide active push-off power during walking. Thus, the present results demonstrate that the semi-active ankle can function like a powered ankle prosthesis only when relatively low positive power is required.

The proposed semi-active ankle prosthesis can function as a passive prosthesis. Our semi-active ankle in *Passive* mode showed a torque profile similar to that of the participant’s passive prosthesis. Interestingly, our semi-active ankle also exhibited a lower dorsiflexion angle than the participant’s passive prosthesis, aligning more closely with the reference ankle angle profile from non-amputee datasets. Notably, the passive behavior of the prosthesis depends on the physical spring in the semi-active ankle actuator and the specific mechanism’s dimensions. Adapting the passive prosthesis behavior to different design targets would require either changing the spring stiffness or the mechanism dimensions. The peak dorsiflexion angle measured during passive walking does not fully match the non-disabled reference data. This discrepancy is due to the spring in the semi-active ankle prosthesis being designed for an 80 kg individual, whereas the participant weighed 72.5 kg. As a result, the spring stiffness implemented in the semi-active ankle was too high for this participant, leading to a reduced peak dorsiflexion angle during passive walking. Despite this discrepancy, the semi-active ankle achieved a peak dorsiflexion angle closer to the non-disabled reference data than the participant’s daily passive ankle/foot prosthesis.

The proposed semi-active ankle prosthesis can transition between *Passive* and *Active* modes quickly enough to enable seamless transitions between level ground and stairs. By using the same control logic previously validated in a unified controller for powered prostheses [48], the proposed high-level controller can detect transitions between these activities in a timely manner. The brake mechanism can engage or disengage and switch between *Active* and *Passive* modes without requiring the user to pause. Specifically, our results show that the brake disengagement time (214 ms) is sufficiently short for the SEA to actively dorsiflex the ankle before the stance phase begins during the transition from walking to stair ascent. Moreover, for our user’s preferred walking speed, our data shows that the brake engagement time is sufficiently short for the semi-active ankle to transition to passive mode before rollover. Therefore, although the brake engagement/disengagement time could be further reduced by supplying higher voltage to the brake motor, it does not seem to be necessary.

Ramp adaptation has been shown to mitigate socket discomfort on ramps [55]. Our results show that the proposed semi-active ankle allows for ramp adaptation. During the terrain-adaptation transition, the participant walked on level ground and ramps using a controller that started in Active mode at heel strike and transitioned to Passive mode after the ankle conformed to the incline of the ground. By transitioning back to *Active* mode at toe-off, the ankle joint can return to a neutral position, thereby making each step independent of the previous one and, in theory, allowing adaptation to rough terrain. However, further studies are necessary to assess whether this prosthesis’s functionality provides tangible clinical benefits for below-knee amputees.

The current implementation of the semi-active ankle prosthesis has some limitations. Because the current prototype was developed to work in combination with an active knee prosthesis, the battery is not integrated into its design. Therefore, using this device with individuals with below-knee amputation would require increasing the device’s weight and possibly the build height. Another limitation specific to our validation protocol is that the stiffness of the proposed semi-active ankle is substantially higher than that of the participant’s prescribed passive prosthesis. In the future, this limitation could be addressed by using a more compliant spring, which we expect will increase ankle range of motion in *Passive* mode, matching passive prostheses. However, a more compliant spring would likely result in a lower open-loop torque-control bandwidth of the SEA in *Active* mode. By design, the proposed semi-active ankle cannot support high positive power tasks, such as powered push-off. Although fully powered operation could be achieved with a larger motor and batteries, this change would increase the device’s weight and size, highlighting the inherent trade-off between functionality and weight.

The proposed semi-active ankle is about 200g lighter than previous fully powered prostheses, such as the Utah Bionic Leg Ankle. Future studies should assess whether this 10–15% mass reduction provides a tangible benefit to users, justifying the absence of powered push-off and net positive energy injection. These future studies should also assess usability in the real world, noting that the proposed semi-active ankle allows safe standing and walking, and performing other activities even when the batteries are fully depleted, which is not possible with other lightweight fully powered prostheses. The proposed semi-active ankle represents a tradeoff between a passive device and a fully powered one. Therefore, future clinical studies are necessary to thoroughly assess the performance of the proposed semi-active device against both passive prostheses and fully powered ones across different modes of operation. These studies should more thoroughly quantify differences in ankle angle and torque trajectories between the proposed semi-active, passive, and active devices and those of non-amputee individuals, including deviations in peak timing and RMSE.

## IX. Conclusion

Passive ankle prostheses are lightweight and compact, but they are optimized only for walking and perform poorly in tasks requiring ankle articulation, such as stair ascent and descent or ramps. In contrast, powered prostheses excel at activities that require ankle articulation but are large and heavy, and unusable when the battery is depleted. In this work, we show that a semi-active ankle prosthesis, combining a series elastic actuator and a braking mechanism, can provide both active ankle articulation during low-power activities (*Active* mode) and passive indefinite support during high-torque activities (*Passive* mode), even when the battery is depleted. The brake enables fast transitions between *Active* and *Passive* modes, allowing for continuous slope adaptation (*terrain adaptation*) and seamless transitions between stairs and level ground (*walk-stair*). To the best of our knowledge, this combination of functionalities has never been shown in semi-active prostheses. Future work should validate the proposed semi-active ankle with amputee users to assess potential clinical benefits compared to passive and powered prostheses.

## Supplementary Material

supp4-3713999

supp2-3713999

supp3-3713999

supp1-3713999

This article has supplementary downloadable material available at https://doi.org/10.1109/TMRB.2026.3713999, provided by the authors.

## Figures and Tables

**Fig. 1.**
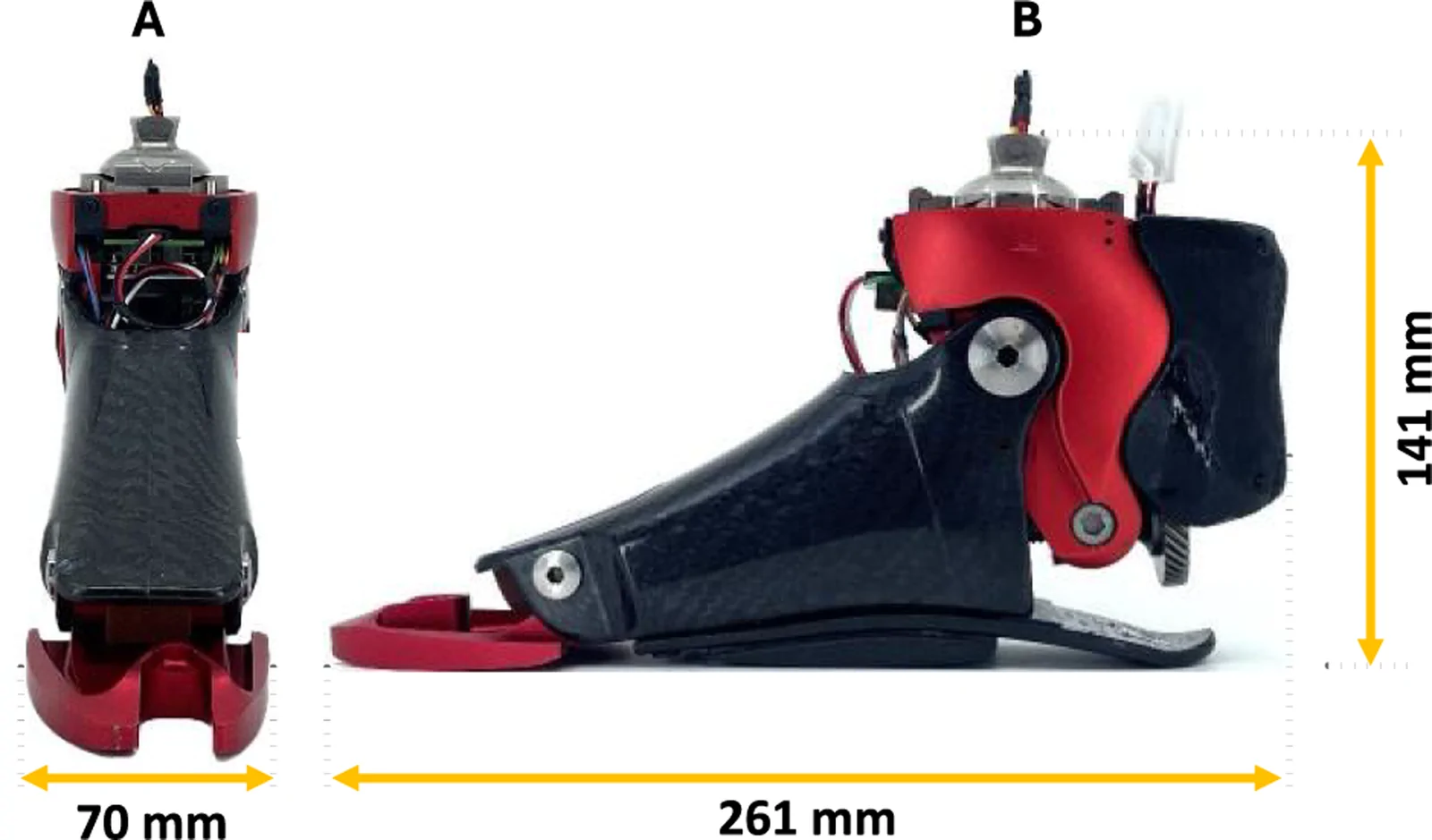
(A) Front-view photo of assembled prosthesis. (B) Side-view photo of assembled prosthesis without the battery.

**Fig. 2.**
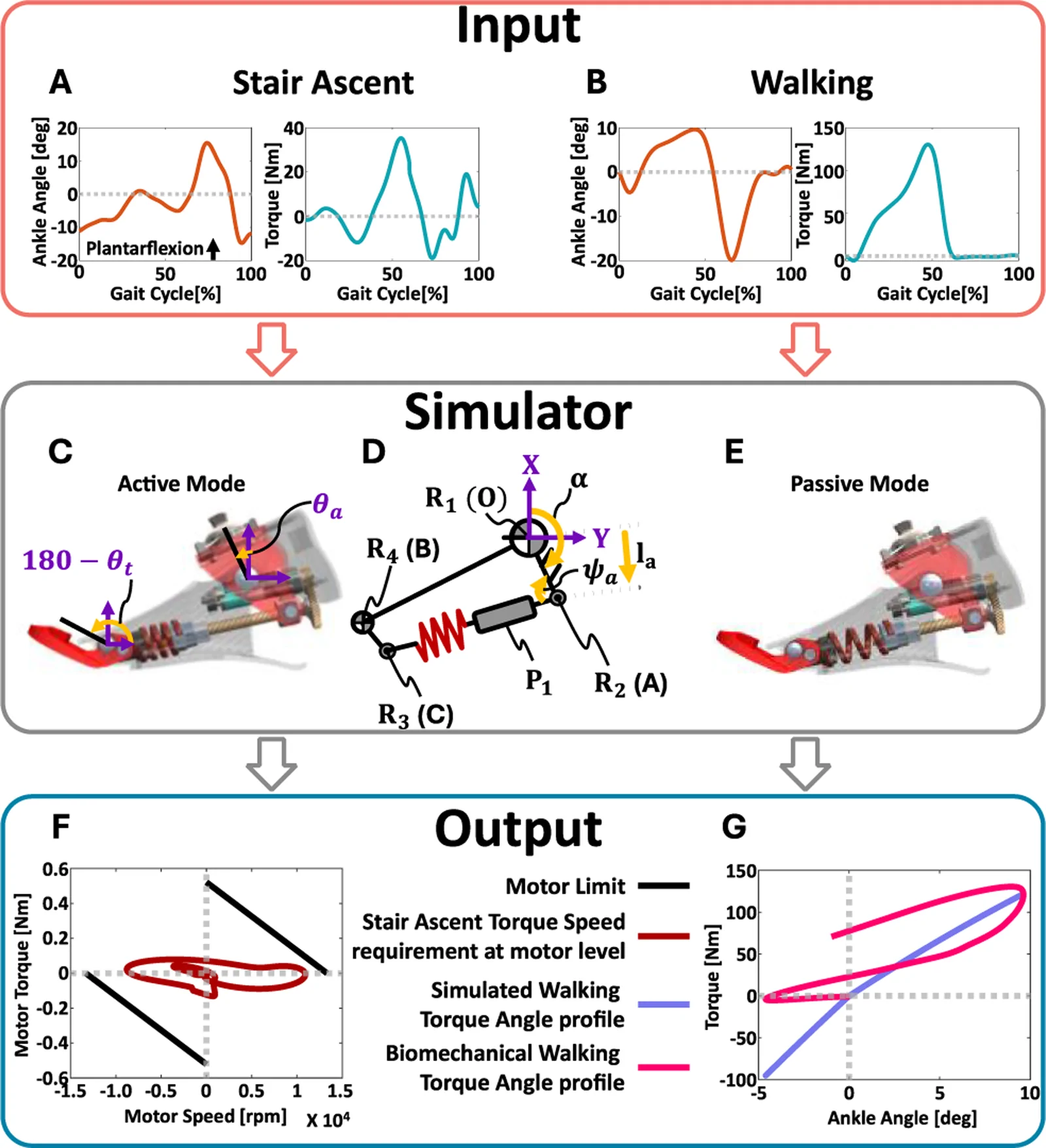
(A) Stair ascent biomechanical data inputs to Active mode simulation. (B) Walking biomechanical data inputs to Passive mode simulation. (C) Semi-active ankle prosthesis in Active mode showing dorsiflexion position through the movement of the motor and ball screw. (D) Semi-active ankle prosthesis kinematic layout. (E) Semi-active ankle prosthesis in Passive mode showing dorsiflexion position through spring deflection. (F) The torque/speed plot of the motor output from the Active mode simulation plotted with the transformed motor level biomechanical data. (G) The torque angle relationship of the ankle from Passive mode simulation plotted with biomechanical walking data.

**Fig. 3.**
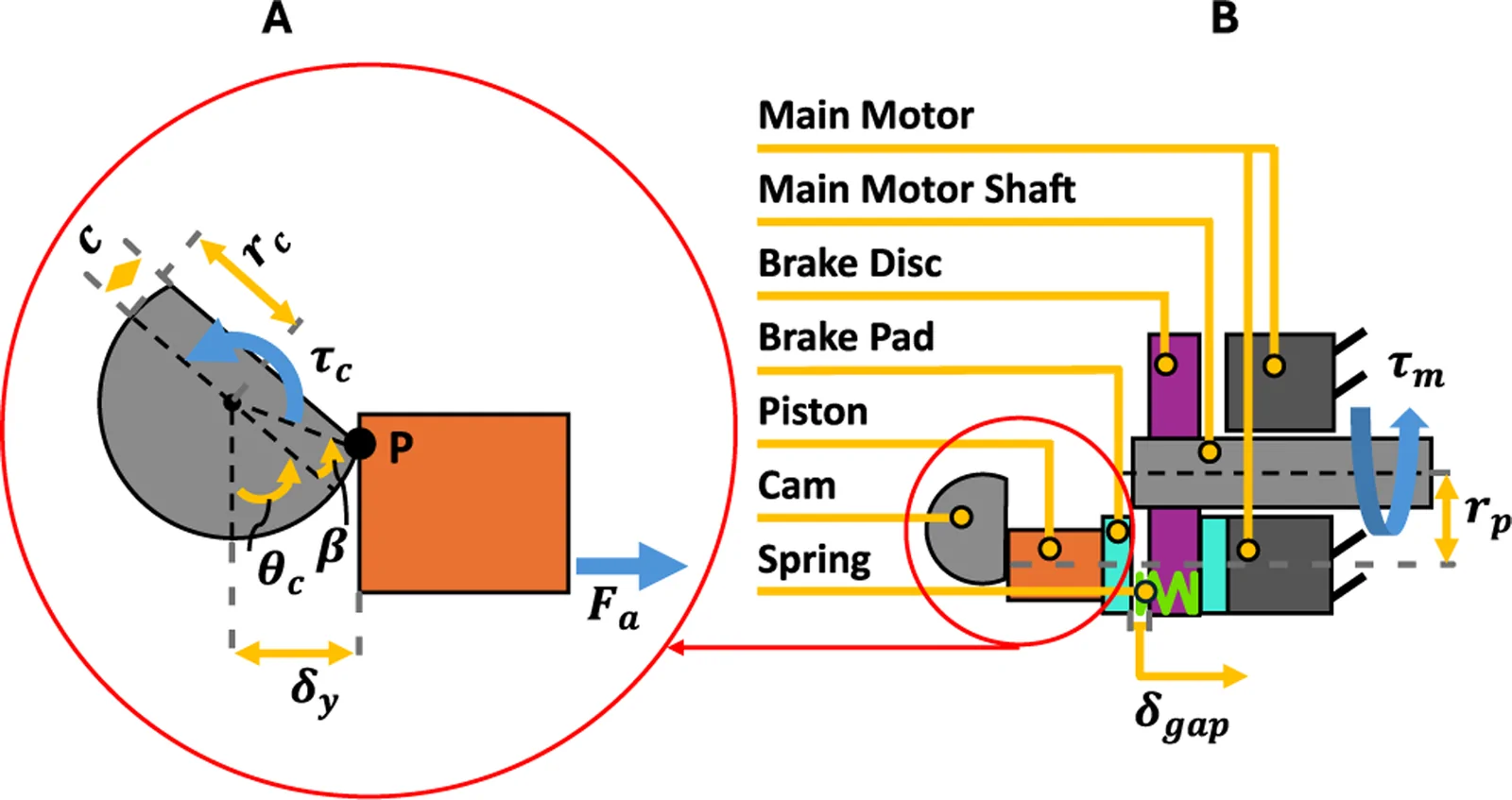
(A) Animated cross-sectional view of brake mechanism with parameter description. (B) View of cam and piston mechanism in disengaged configuration.

**Fig. 4.**
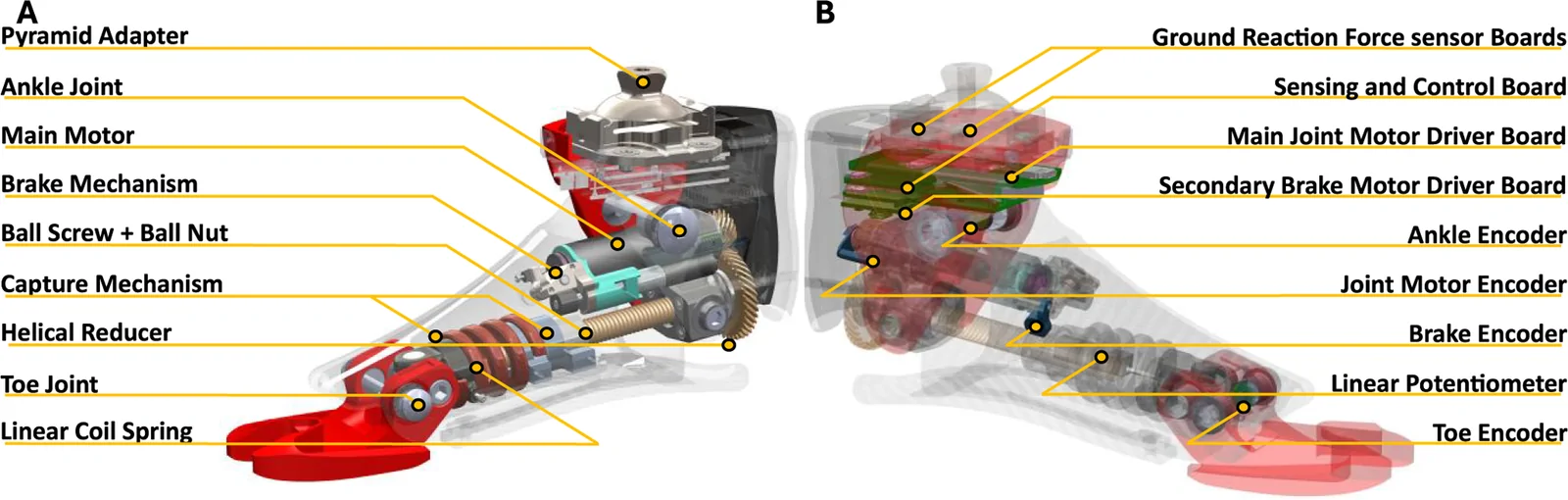
Semi-active ankle prosthesis overview. (A) Main mechanical components. (B) Main electrical components.

**Fig. 5.**
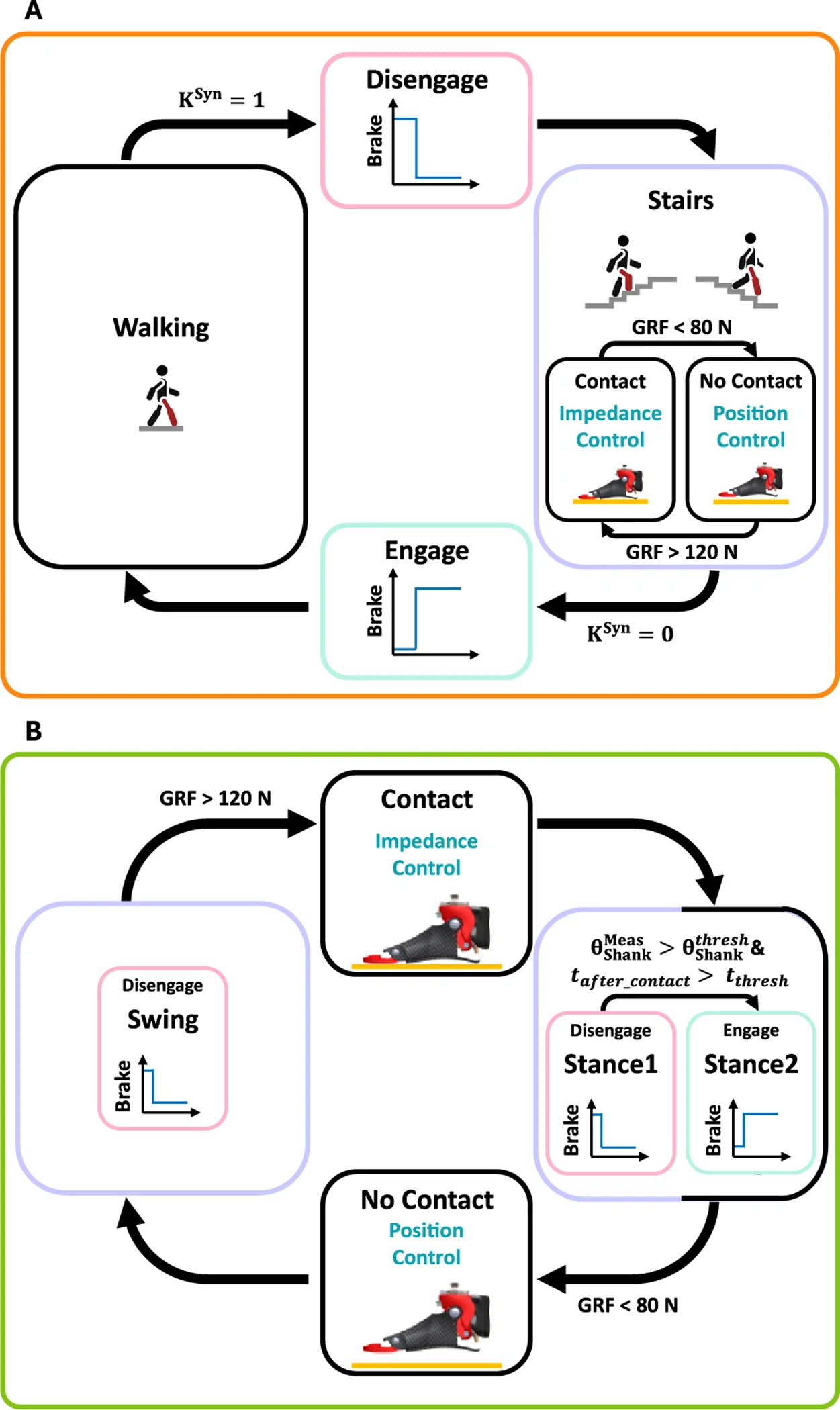
Controller. (A) *Walk-Stair transition* controller scheme. (B) *Terrain-adaptation* controller scheme.

**Fig. 6.**
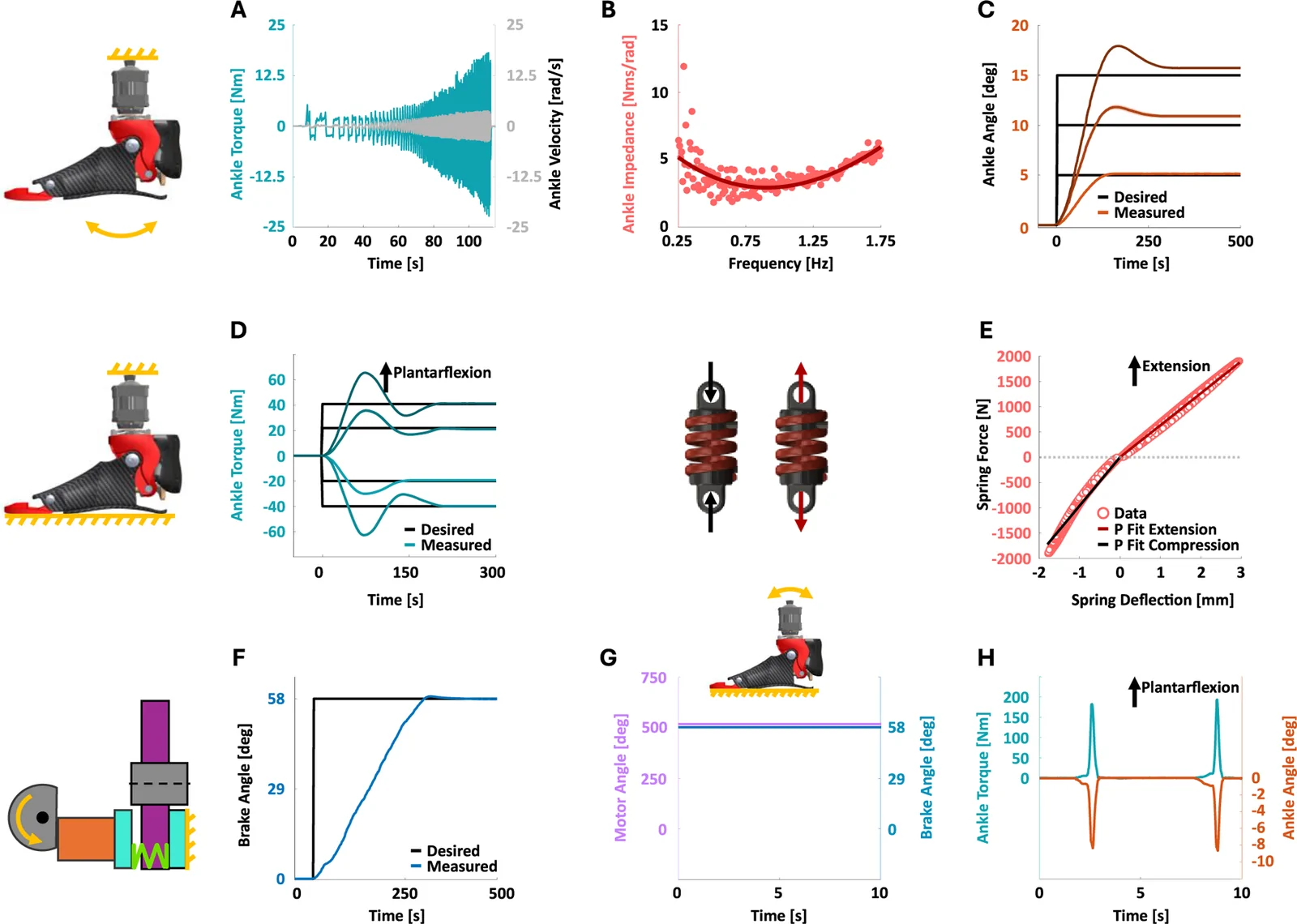
Results from Benchtop Testing. (A) Ankle backdriving torque and estimated impedance when backdriven at various frequences. (B) Impedance and third order polynomial fitted model of the ankle in the frequency domain. (C) Ankle joint desired and measured closed-loop position step response. (D) Ankle joint desired and measured open-loop torque step response. (E) Spring characterization and linear fitted model showing different stiffnesses in tension and compression. (F) Brake desired and measured closed-loop position step response. (G) Motor Angle and Brake Angle during brake holding torque test in passive mode. (H) Demonstration of brake holding torque at the ankle joint in passive mode.

**Fig. 7.**
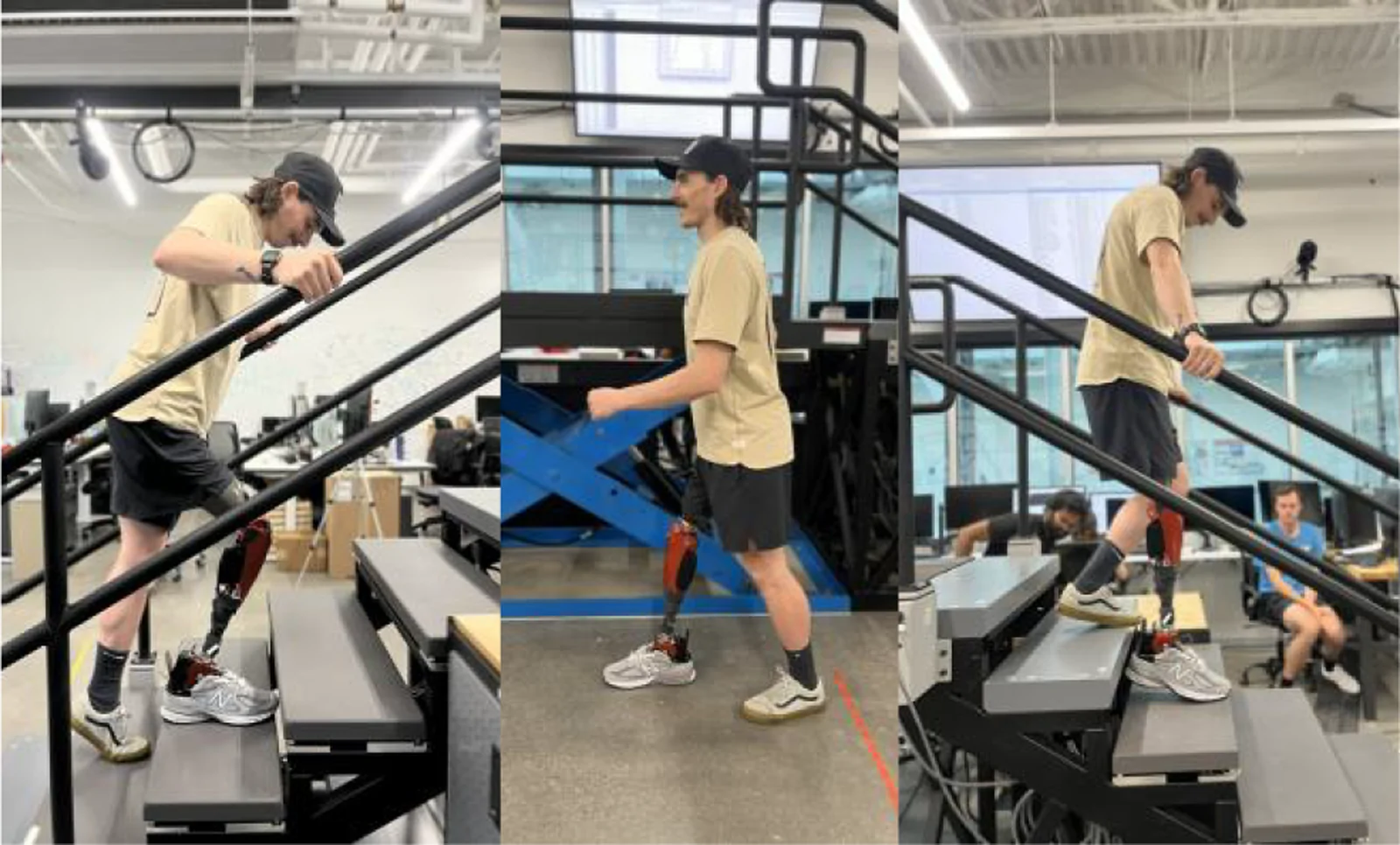
Participant wearing the semi-active ankle prosthesis. Left: Participant ascending instrumented stairs with the powered knee prosthesis and semi-active ankle prosthesis in Active Mode. Middle: Participant walking with the powered knee prosthesis and semi-active ankle prosthesis in Passive Mode. Right: Participant descending instrumented stairs with the powered knee prosthesis and semi-active ankle prosthesis in Active Mode.

**Fig. 8.**
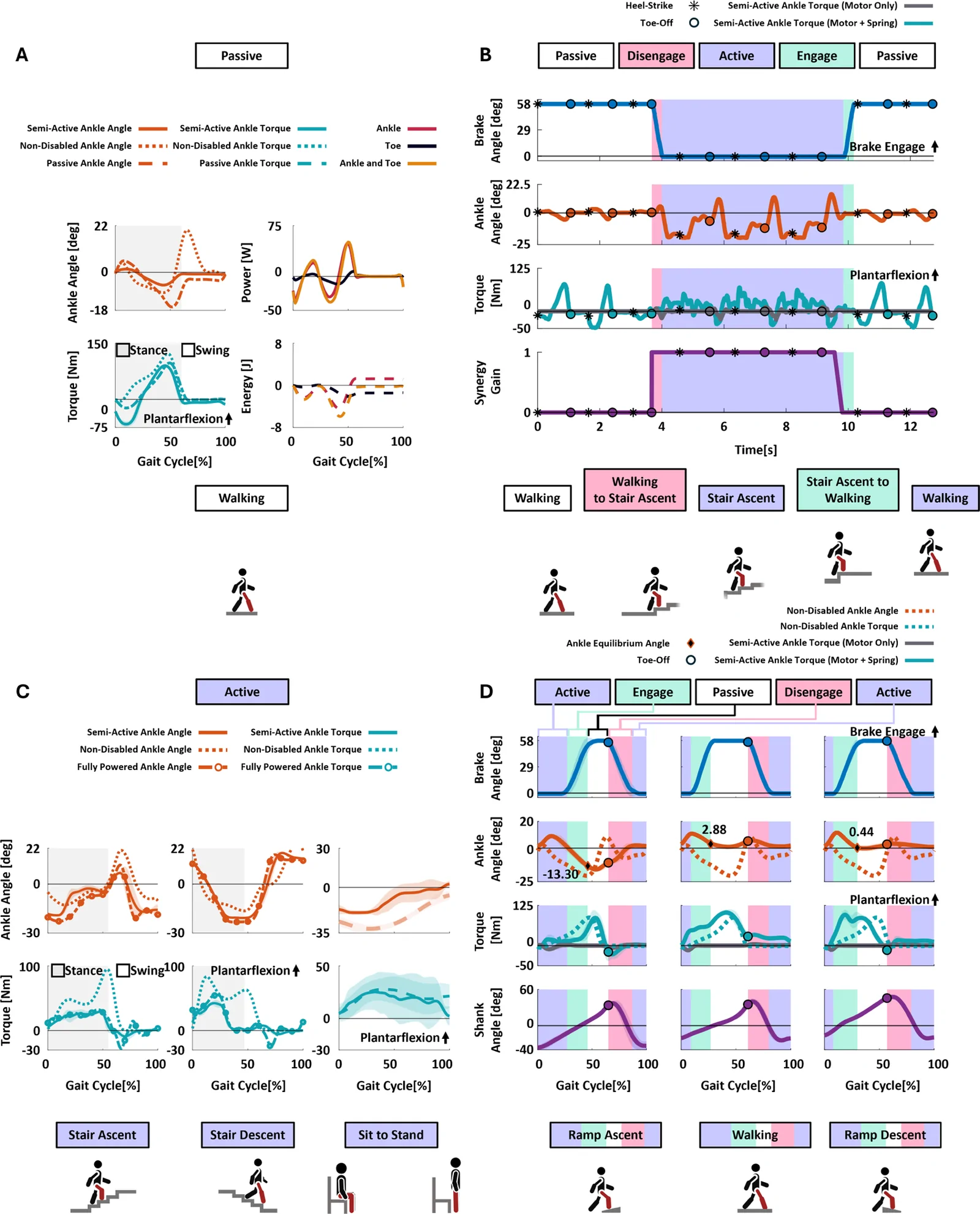
Results from human subject testing. (A) Kinematic and Kinetic data from one amputee participant walking on the semi-active ankle in Passive mode averaged over 15 strides. (B) Kinematic and Kinetic data from one amputee participant during automatic walk-stair transitioning controller using with the semi-active ankle. C) Kinematic and Kinetic data from one amputee participant performing stair ascent and descent and sit-to-stand transition on the semi-active ankle in Active mode, averaged over 7 strides. (D) Kinematic and Kinetic data from one amputee participant performing ramp ascent, level-ground walking, and ramp descent on the semi-active ankle during automatic terrain-adaptation controller, averaged over 8 strides.

**TABLE I:** Semi-Active Ankle Design Objectives

Parameter	Desired	Actual

Range Of Motion (DF /PF) [deg]	11 / 31	26 / 24
Peak Passive Torque [Nm]	127	150
Device Weight [kg]	1.5	1.4
Peak Power [W]	140	115
Build Height [mm]	142	141

**TABLE II:** Comparative Analysis

Devices	Build Height [mm]	Weight [kg]	ROM (DF – 0 – PF) [deg]	Device Functionality	Peak Torque [Nm]^a^	Active Motor Power (W)	Functionality after battery depletion^a^

**Semi Active Ankle [This Paper]**	141	1.4^b^	−26 – 0 – 24	Semi-Active	150^c^/55	115	Y
Utah Bionic Leg - Ankle [15]	179	1.6	−20 – 0 – 20	Powered	125	200	N
Utah Polycentric Ankle [18]	120	1.3^b^	−28 – 0 – 27	Powered	125	200	N
Ottobock Empower/BIOM [20]	221^d^	2.2	−10 – 0 – 24	Powered	125	200	Y^e^
RFSEA Ankle [17]	223	2.0	−21 – 0 – 40	Powered	160	350	N
VL (Gen 3) – Ankle [52]	210	2.3	−30 – 0 – 45	Powered	140	200	N
LI – Ankle [13]	250^f^	3.0^f^	−45 – 0 – 45	Powered	130	410	N
OSL (V1) – Ankle [12]	213	1.7	30^g^	Powered	178	750	N
AMP foot [56]	200	2.5^b^	−15 – 0 – 30	Powered	125	60	Y
AMP Foot 4+ [57]	260	2.3^b^	−15 – 0 – 40	Semi-Active	-	200	Y
Bartlett [32]	163	1.35	−12 – 0 – 30	Semi-Active	150^h^	10	Y
VSPA [27]	118	1.1	−30 – 0 – 30	Semi-Active	150^h^	10	Y
Ottobock Meridium [33], [41]	166	1.5	−14.5 – 0 – 22	Semi-Active	-	-	Y
Ossur Proprio [26], [58]	180	1.5	33^g^	Semi-Active	-	-	Y
VSF [31]	87	0.65	33^g^	Semi-Active	-	1.6	Y
Non-backdrivable RAP [28]	50	1.2^b^	−20 – 0 – 20	Semi-Active	94^h^	12	Y
Ottobock Taleo [4]	155	0.5	-	Passive	150^h^	-	Y

a Research informed analysis of devices.

b Devices with offboard battery.

c Passive Peak Torque.

d Reported with foot shell.

e Partial battery empty functionality.

f Half the reported parameters.

g Reported one value for ROM.

h Only Passive Torque.

Y – yes, N – No.
