# Supplementary material for: A Semi-Active Ankle Prosthesis With Automatic Transitions Between Passive and Active Modes
Source: IEEE Trans Med Robot Bionics. Author manuscript; Available in PMC 2026 Nov 5. (PMC13733658; doi:10.1109/tmrb.2026.3713999)
Supplement: Supplementary file 1 [file NIHMS2204911-supplement-supp4-3713999.pdf]

Supplementary Materials for:

A Semi-Active Ankle Prosthesis with Automatic Transitions Between Passive and Active Modes

Contents:

I. SUPPLEMENTARY

S1, Brake Overview

S2, Electrical Architecture

S3, Mid-level Controller

S3a, Series Elastic Actuator Mid-level Controller

S3b, Brake Mid-level Controller

S4, Unified high-level Impedance Controller Summary

S5, Unified high-level Position Controller Summary

S6, Additional Amputee Testing

S6a, Stand to Sit

S6b, Passive Mode across activities

I. SUPPLEMENTARY

S1, Brake Overview

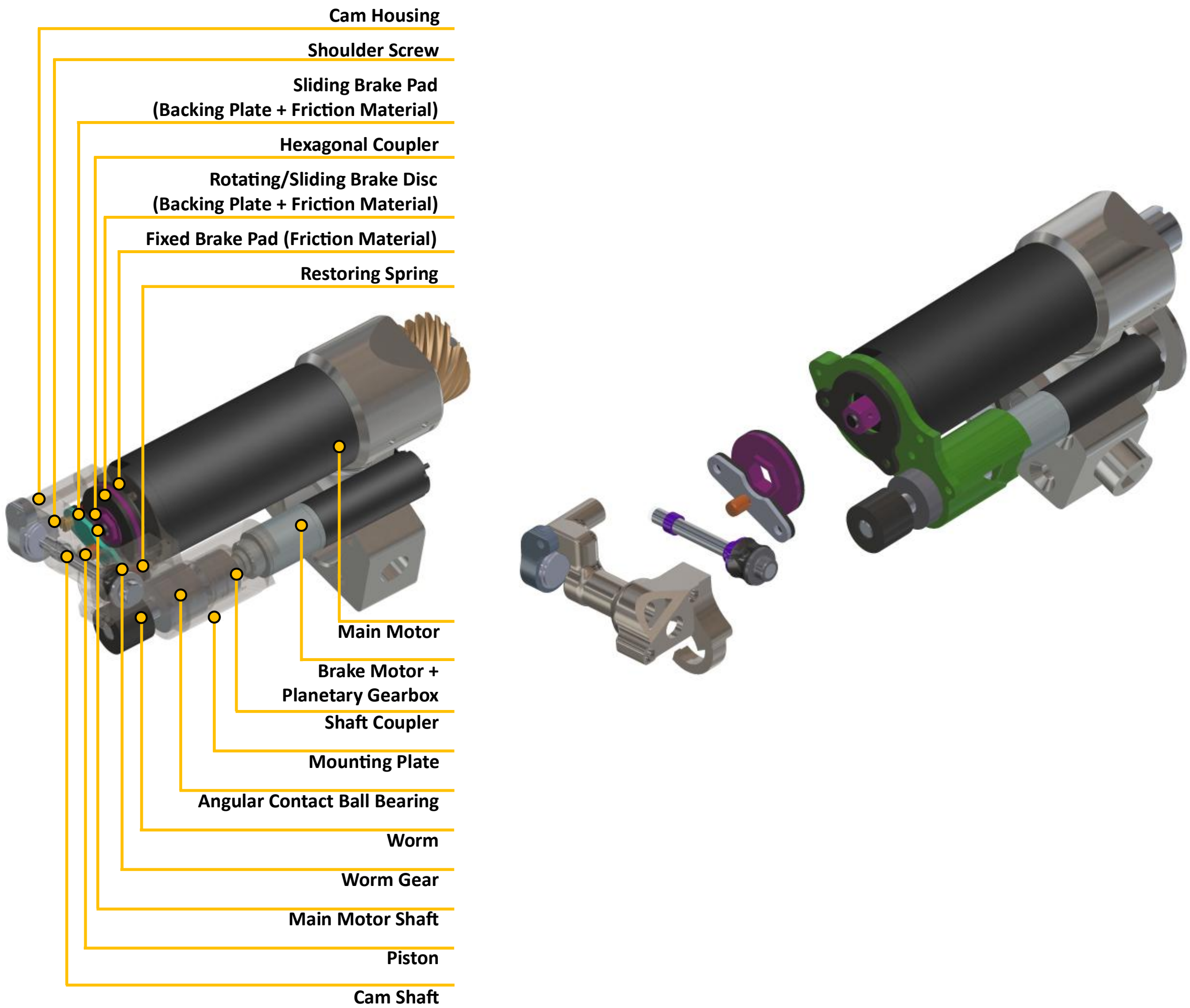

Fig.S1. Brake overview: Components in brake mechanism, Components in cam and piston mechanism, components in worm drive (non-backdrivable mechanism), components in geared brake motor, components used for mounting the brake to the main joint motor

Fig.S1. shows the location of the brake mechanism relative to the Main Motor (Maxon EC22 4-pole, 24 V, 90 W) of the series elastic actuator in the semi-active ankle prosthesis. The brake motor (Maxon EC10 brushless motor, 18 V, 8 W) is coupled to a 16:1 planetary gearbox (Maxon GP10 16:1). The planetary gearbox drives a worm through a flexible shaft coupler. The worm wheel, which is driven by the worm, is pressed into a cam shaft that linearly displaces a piston. The movement of the piston pushes the sliding brake pad consisting of a brake pad liner (McMaster 60895K11) and a hardened 440c stainless steel backing plate which in turn presses the brake disc against the fixed brake thereby arresting the main motor shaft, which is attached to the brake pad through a hexagonal coupler. When the brake disengages, the brake motor spins in the counterclockwise direction, which releases the pressure on the brake piston. This allows the restoring spring to push the sliding brake pad away from the brake disc, thus allowing free rotation of the main motor.

S2, Electrical Architecture

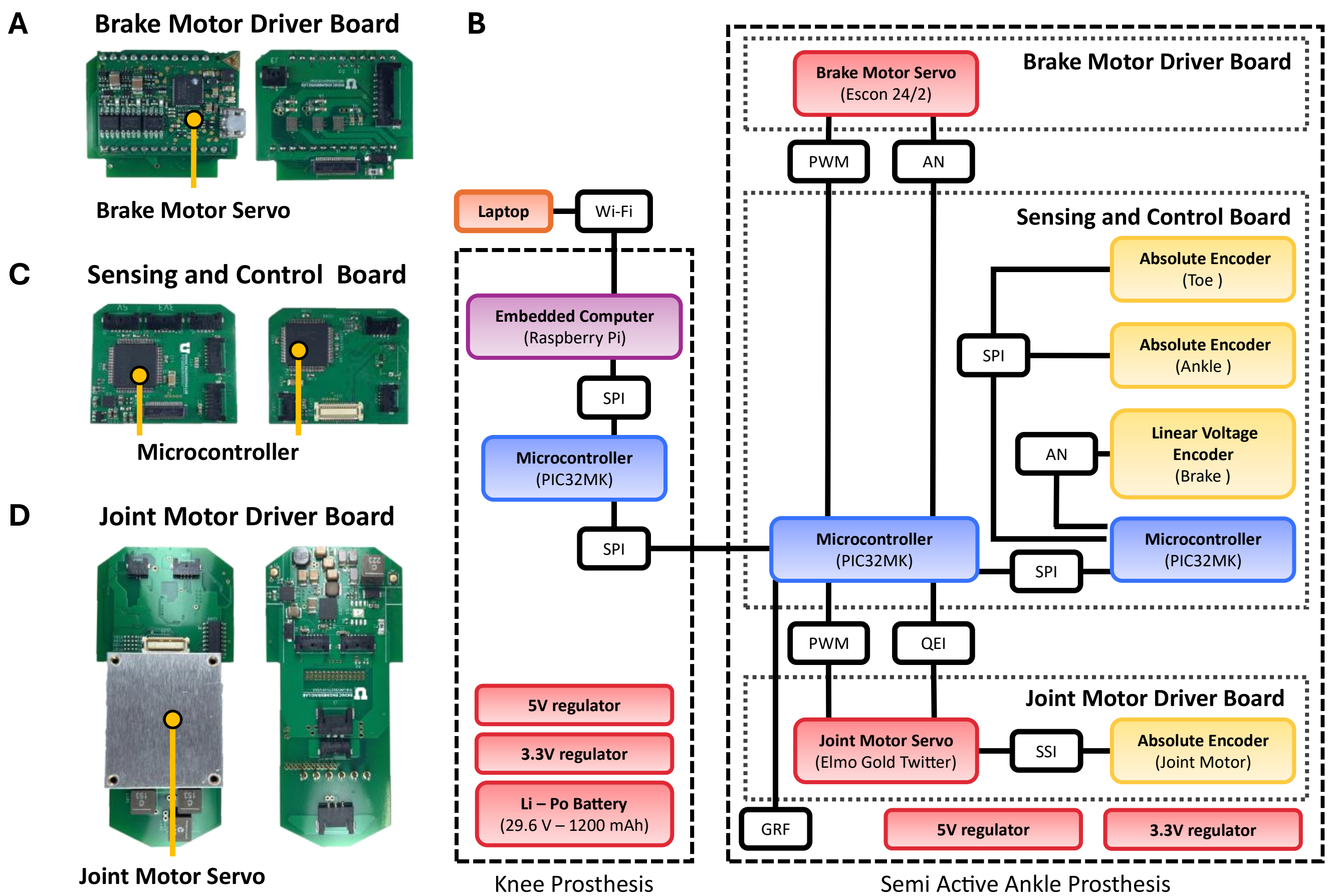

Fig.S2. Electrical Architecture. (A) Brake motor driver board. (B) Sensing and control board. (C) Joint motor driver board. (D) Schematic architecture of electrical system.

Fig.S2. shows the electronics housed in the semi-active ankle prosthesis and its communication protocol with the knee prosthesis. The Brake motor driver board, the sensing and control board and joint motor driver board are stacked together on the shank frame of the semi-active ankle and communicate with the knee prosthesis through SPI communication. The ankle electronics share battery power with the knee prosthesis. The joint motor servo in the joint motor driver board performs closed loop current control on the Maxon motor in the ankle joint, and the brake motor driver performs closed loop current control on the brake motor. Both motor drivers are given current commands using PWM signals from the PIC microcontroller. The mid-level controller is executed on a PIC32 microcontroller in the sensing and control board and communicates bidirectionally with another PIC32 in the sensing and control board using SPI communication. The second PIC32 handles gathering and processing sensing signals from motor, joint and brake encoders.

S3, Mid-level Controller

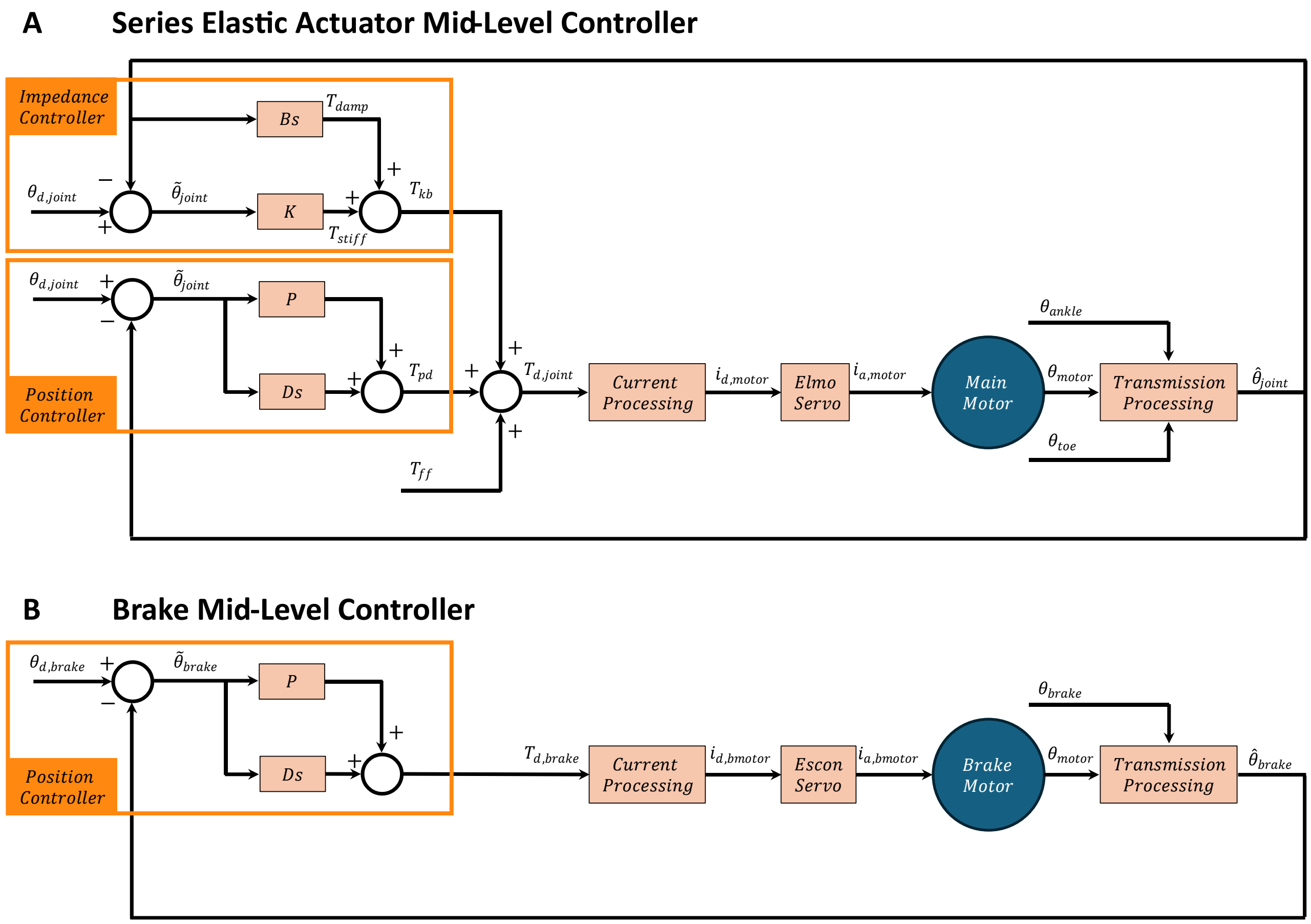

Fig.S3. Block Diagram for mid-level control. (A) Series Elastic Actuator mid-level controller highlighting impedance and position controllers. (B) Brake mid-level controller highlighting position controller.

Fig.S3. shows the mid-level controller of the series elastic actuator (A) and the brake motor (B). The series elastic actuator has two mid-level controllers (Impedance and Position controller) which are chosen based on the contact/no-contact condition from the high-level controller. The Impedance and Position controllers receive desired joint position ($\theta_{d,joint}$) from the high-level unified controller. Based on the calculated kinematic joint position ($\hat{\theta}_{joint}$), calculated from ankle, toe, motor encoders and kinematics, an error signal ($\tilde{\theta}_{joint}$) is generated. Based on the contact/no-contact condition, the joint torques can be T_{kb} or T_{pd}. These torques are added to calculate the desired joint torque ($T_{d,\,joint}$). Using the desired joint torque, transmission ratio, and motor torque constant, a desired motor current ($i_{d,\,motor}$) is calculated. The desired motor current is used by the Elmo servo to control the main motor.

The brake motor is controlled using a position controller. The desired brake position ($\theta_{d,brake}$) switches between 0 and 58 degrees, where 58 degrees denotes the fully engaged brake position. Based on the high-level controller, a boolean on/off value is sent to the position controller which commands the brake to engage or disengage. The calculated brake position ($\hat{\theta}_{brake}$) is used to create an error signal ($\tilde{\theta}_{brake}$) and a PD controller calculates a desired total brake torque is ($T_{d,\,brake}$). Using $T_{d,\,brake}$, brake transmission ratio and brake motor constant a desired brake motor current ($i_{d,\,bmotor}$) is calculated. The Escon servo uses this desired motor current to drive the brake motor.

S4, Unified high-level Impedance Controller Summary

Impedance Control

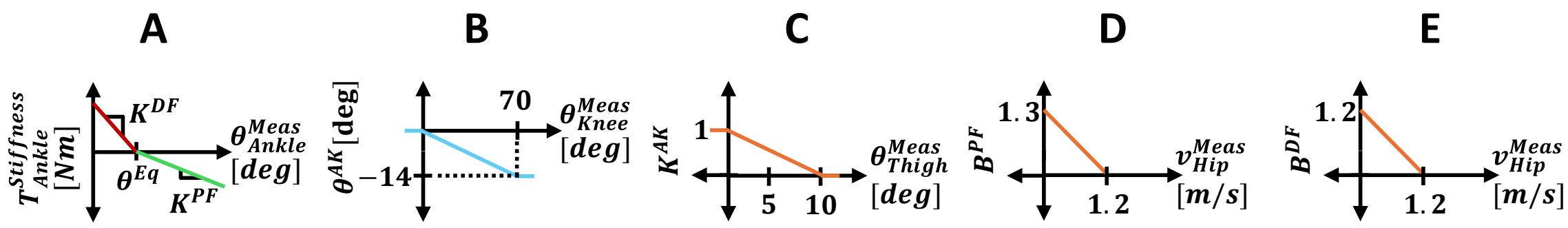

$$T_{Ankle} = T_{Ankle}^{Stiffness} + T_{Ankle}^{Damping}$$

$$T_{Ankle}^{Stiffness} = K^{Stiffness} * (\theta_{Ankle}^{Meas} - \theta^{Eq}) \begin{cases} K^{Stiffness} = \begin{cases} K^{DF} = 4, if \theta_{Ankle}^{Meas} < \theta^{Eq} \\ K^{PF} = 1, if \theta_{Ankle}^{Meas} \geq \theta^{Eq} \end{cases} \\ \theta^{Eq} = \theta^{Eq1} + \theta^{Eq2} \\ \theta^{Eq1} = min\left(\left(\theta_{Ankle}^{Meas} + \theta_{Shank}^{Meas}\right), \pm 8\right) \\ \theta^{Eq2} = K^{AK} * \theta^{AK} \end{cases}$$

$$T_{Ankle}^{Damping} = -B^{Damping} * \dot{\theta}_{Ankle}^{Meas} \begin{cases} B^{Damping} = \begin{cases} 1.3, \ if \theta_{Shank}^{Meas} < 0 \\ B^{PF}, \ if \dot{\theta}_{Ankle}^{Meas} \geq \theta^{Eq} \\ B^{DF}, \ if \dot{\theta}_{Ankle}^{Meas} < 0 \end{cases} \\ v_{Hip}^{Meas} = \sin(\alpha + \theta_{Foot}^{Meas}) * l_{Foot}^{Meas} * \dot{\theta}_{Foot}^{Meas} \\ \quad + \cos(\theta_{Shank}^{Meas}) * l_{Shank}^{Meas} * \dot{\theta}_{Shank}^{Meas} \\ \quad + \cos(\theta_{Thigh}^{Meas}) * l_{Thigh}^{Meas} * \dot{\theta}_{Thigh}^{Meas} \end{cases}$$

B^{PF} *and* B^{DF} *are defined using* v_{Hip}^{Meas}

where,
T_{Ankle} = *Total ankle torque during contact*
$T_{Ankle}^{Stiffness}$ = *Virtual stiffness*
$T_{Ankle}^{Damping}$ = *Virtual damping*
θ_{Ankle}^{Meas} = *Current ankle angle*
$\dot{\theta}_{Ankle}^{Meas}$ = *Current ankle velocity*
θ^{Eq} = *desired equilibrium angle*
$K^{Stiffness}$ = *Stiffness gain based on* θ^{Eq}
θ^{AK} = *Ankle knee synergy angle*
K^{AK} = *Ankle knee synergy gain*
$B^{Damping}$ = *Damping gain based on* θ_{Shank}^{Meas}, $\dot{\theta}_{Ankle}^{Meas}$
v_{Hip}^{Meas} = *Horizontal Hip velocity*
θ^{AK} = *Ankle knee synergy angle*
K^{AK} = *Ankle knee synergy gain*

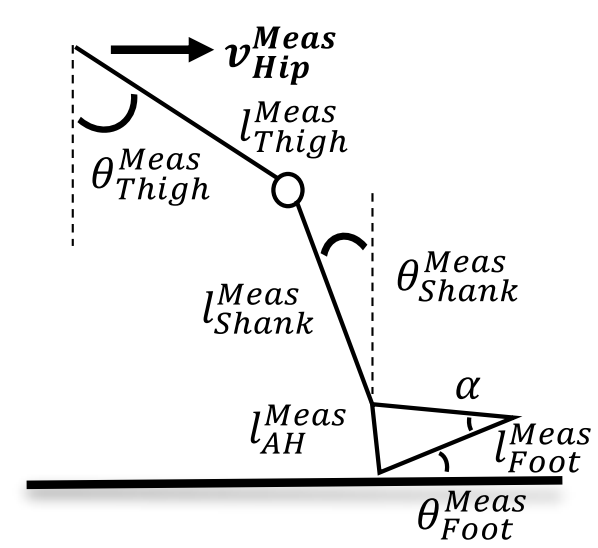

Fig.S4. Unified high-level impedance controller summary.

During *contact* condition, a high-level impedance controller (Fig.S4) is used to define the desired set-point (θ^{Eq}) and impedance ($K^{Stiffness}, B^{Damping}$) parameters at the ankle. The desired set-point (θ^{Eq}) is a two-part equation (θ^{Eq1}), (θ^{Eq2}). θ^{Eq1} is a function of current ankle angle (θ_{Ankle}^{Meas}), and current shank angle (θ_{Shank}^{Meas}), which is calculated from the current thigh angle (θ_{Thigh}^{Meas}) from the IMU on the knee prosthesis and knee angle (θ_{Knee}^{Meas}). θ^{Eq2} is a function of K^{AK} and θ^{AK} which are variables defined by current knee angle (θ_{Knee}^{Meas}) and current thigh angle (θ_{Thigh}^{Meas}) using predefined thresholds as shown in the subplot B and C. $K^{Stiffness}$ is defined by the (θ_{Ankle}^{Meas}) and calculated (θ^{Eq}), while $B^{Damping}$ is defined by (θ_{Shank}^{Meas}), ($\dot{\theta}_{Ankle}^{Meas}$) and ($\theta^{Eq}$) as shown in the above equations.

S5, Unified high-level Position Set-Point Controller Summary

Position Control

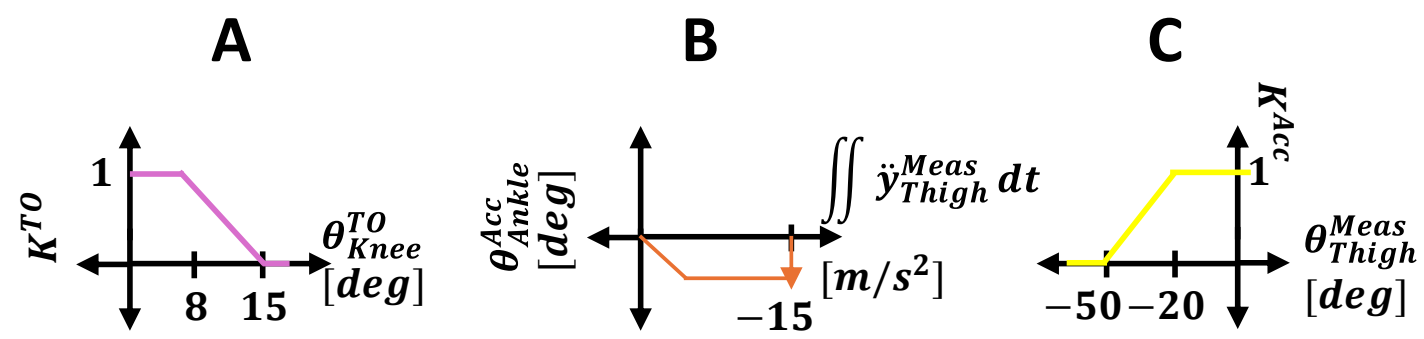

$$T_{Ankle} = T_{Ankle}^{Proportional} + T_{Ankle}^{Derivative}$$

$$T_{Ankle}^{Proportional} = K^P * (\theta_{Ankle}^{Des} - \theta_{Ankle}^{Meas}) \begin{cases} \theta_{Ankle}^{Des} = \theta_{Ankle}^{Syn} * K^{Syn} \\ \theta_{Ankle}^{Syn} = \theta_{Ankle}^{Pos} + \theta_{Ankle}^{Acc} \begin{cases} \theta_{Ankle}^{Pos} = K^{AK} * \theta^{AK} \\ \theta_{Ankle}^{Acc} = K^{Acc} * 1000 * \iint (\ddot{y}_{Thigh}^{Meas} - 1.03)dt \end{cases} \\ K^{Syn} = max\left(min\left((K^{TO} + \Delta K), 1\right), 0\right) \\ \Delta K = \begin{cases} 0.016, if\ \theta_{Thigh}^{Meas} < -35\ \&\ \dot{\theta}_{Thigh}^{Meas} < -20\ \&\ \theta_{Knee}^{Meas} > 20 \\ -0.008, if\ -10 > \theta_{Thigh}^{Meas}\ \&\ \theta_{Thigh}^{Meas} > -35\ \&\ \dot{\theta}_{Thigh}^{Meas} > -50 \end{cases} \end{cases}$$

$$T_{Ankle}^{Derivative} = K^D * \dot{\theta}_{Ankle}^{Meas}$$

$K^P and K^D$ are loosely tuned PD gains

where,
T_{Ankle} = Total ankle torque during No contact
$T_{Ankle}^{Proportional}$ = Proportional Term
$T_{Ankle}^{Damping}$ = Derivative Term
θ_{Ankle}^{Meas} = Current ankle angle
$\dot{\theta}_{Ankle}^{Meas}$ = Current ankle velocity
θ_{Ankle}^{Des} = desired ankle angle
θ_{Ankle}^{Syn} = Synergistic ankle angle
θ_{Ankle}^{Pos} = Position based synergy
θ_{Ankle}^{Acc} = Acceleration based synergy
θ^{AK} = Ankle knee synergy angle
K^{AK} = Ankle knee synergy gain
K^{Acc} = Synergy acceleration gain
$\ddot{y}_{Thigh}^{Meas}$ = Vertical acceleration of thigh
K^{Syn} = Synergy gain
K^{TO} = Component of K^{Syn} calculated at toe off
θ_{Knee}^{Meas} = Current knee angle

Fig.S5. Unified high-level position controller summary.

During *no contact* condition, a high-level position controller (Fig.S5) is used to define the desired set-point (θ_{Ankle}^{Des}) and position gain parameters (K^P, K^D) at the ankle. The desired set-point (θ_{Ankle}^{Des}) is a function of (θ_{Ankle}^{Syn}) and (K^{Syn}). (θ_{Ankle}^{Syn}) is a two part equation which consists of (θ_{Ankle}^{Pos}) which is synergistic ankle angle calculated from of K^{AK} and θ^{AK} as stated in the impedance controller and (θ_{Ankle}^{Acc}) which is calculated from of K^{Acc} as defined in subplot C and ($\ddot{y}_{Thigh}^{Meas}$) which the vertical acceleration of the thigh. (K^{Syn}) is calculated using knee angle at toe off (θ_{Knee}^{TO}) as shown in subplot A and (ΔK) calculated using current thigh angle (θ_{Thigh}^{Meas}), current thigh velocity ($\dot{\theta}_{Thigh}^{Meas}$) and current knee angle (θ_{Knee}^{Meas}). (K^P, K^D) gains are loosely tuned.

S6, Additional Amputee Testing

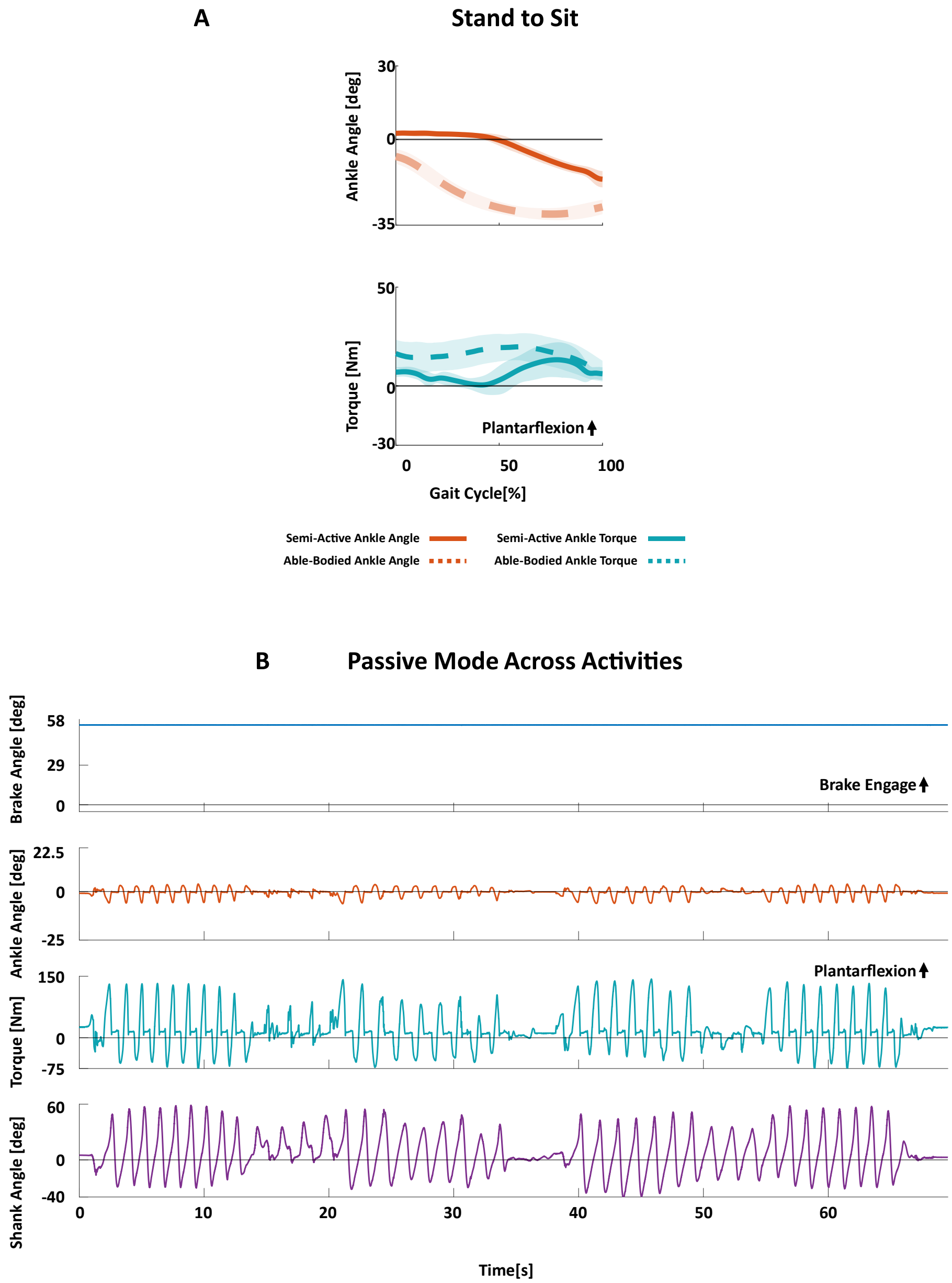

Fig.S6. Additional Amputee Testing Data. (A) Sit to Stand. (B) Passive Mode ambulation across activities using the Semi-Active Ankle.

In stand to sit transition the peak dorsiflexion angle was 16º and peak torque of 12 Nm for the Semi-Active Ankle in the Active mode. The able-bodied dataset reached 30º of dorsiflexion and 19 Nm of peak torque.

In passive mode the user was able to ambulate across all activities using the semi-active ankle highlighted by the brake angle. The semi-active ankle in this mode functions as a heavy passive ankle allowing the user to ambulate across activities while providing assistance from the displacement of the linear spring.
